# Integrated experimental and computational characterization of bioactive metabolites from *Streptomyces paradoxus GH53* with antimicrobial, antioxidant, and antitumor activities

**DOI:** 10.1038/s41598-026-62673-2

**Published:** 2026-07-23

**Authors:** Gehad H. El Sayed, Asmaa M. Fahim, Mohamed Fadel, Manal S. Selim, Rasha Fouad, Mohamed E. El Awady

**Affiliations:** 1 Microbial Chemistry Department, National Research Centre, Dokki, Cairo, 12622 Egypt; 2 Department of Green Chemistry, National Research Centre, P.O. Box 12622, Dokki, Cairo, Egypt; 3 Microbial Biotechnology Department, National Research Centre, Dokki, Cairo, 12622 Egypt; 4 Medicinal and Aromatic Plant Research Department, National Research Centre, Dokki, Cairo, 12622 Egypt

**Keywords:** *Streptomyces paradoxus GH53*, Secondary metabolites, GC-MS analysis, Antioxidant activity, Antitumor activity, Antimicrobial activity, Molecular docking, Molecular dynamics simulation, ADMET profiling, Biochemistry, Biotechnology, Chemical biology, Chemistry, Computational biology and bioinformatics, Drug discovery, Microbiology

## Abstract

Streptomyces species are widely recognized as valuable sources of secondary metabolites with diverse biological activities. In this study, the isolate GH53 was identified as Streptomyces paradoxus based on morphological characteristics and partial 16 S rRNA gene sequence analysis. Fermentation conditions were optimized to enhance metabolite production, and the crude ethyl acetate extract was chemically characterized using GC-MS, FT-IR, and UV-Vis spectroscopy. The analytical profile suggested the presence of a chemically complex mixture containing fatty acids, fatty acid derivatives, hydrocarbons, and terpenoid-related constituents. The IC₅₀ values of the crude extract were 0.175 ± 0.013 and 0.097 ± 0.006 mg/mL for the DPPH and ABTS radical scavenging assays, respectively. Because the calculated DPPH IC₅₀ value was slightly below the lowest tested concentration, it should be interpreted as a fitted estimate derived from the dose–response curve rather than as a directly measured concentration point. It also showed cytotoxic activity against HePG-2 and MCF-7 cell lines, with IC₅₀ values of 19.50 ± 1.5 and 28.81 ± 2.0 µg/mL, respectively. To provide a preliminary molecular interpretation of these extract-level bioactivities, selected representative metabolites tentatively identified by GC-MS were evaluated individually as defined ligands using molecular docking, molecular dynamics simulation, and ADMET prediction. The modeled compounds showed favorable predicted interactions with selected antimicrobial-, antioxidant-, and anticancer-related protein targets, and the corresponding protein–ligand complexes generally maintained stable interaction profiles during simulation. However, ADMET analysis indicated potential limitations for some high-molecular-weight lipophilic constituents, including poor drug-likeness, limited predicted solubility, and possible toxicity liabilities. Overall, these findings suggest that S. paradoxus GH53 represents a promising source of bioactive metabolites for future fractionation, purification, structural confirmation, and compound-level biological evaluation. The results should be interpreted as preliminary screening evidence and not as confirmation of therapeutic efficacy or direct systemic drug suitability.

## Introduction

Streptomycete species produce a wide variety of biological compounds that are of great importance to the medical community^1^. To date, over two-thirds of all naturally occurring antibiotics have been derived from Streptomycetes, as well as many other important medicinal compounds, such as those used to treat cancer, fungal infections, inflammation, and also as immunomodulators^2^. The unique ability of Streptomycetes to produce such an extensive range of natural products is because they possess some of the largest genomes of any bacterial species^3^, with many of these genomes containing large numbers of biosynthetic gene clusters (BGCs) that encode the biosynthesis of polyketides, non-ribosomal peptides, terpenoids, and lipid-based metabolites that have been associated with diverse biological activities^4^. As antimicrobial resistance increases and cancer incidence rises, there is a growing need to identify new bioactive compounds^5^. Soil-based Streptomyces species have adapted to many different ecosystems, which allows them to produce a wide variety of compounds with therapeutic potential. The most well-studied of these genera are the Streptomyces strains, which produce extensive libraries of complex mixtures of fatty acids and their ester derivatives^6^.

The Egyptian rhizosphere is a very attractive biological niche for the identification of novel Streptomyces strains due to its influence by several abiotic stresses, such as aridity, significant temperature changes, salinity, limited nutrient availability, and heterogeneous soil chemistry. Adverse environmental conditions can induce microbial adaptation and trigger distinct biosynthetic pathways related to stress resilience and secondary metabolite synthesis. Actinomycetes linked with the rhizosphere encounter significant interactions between plants and microbes, as well as among microbes, which subsequently enhance the production of chemically varied bioactive compounds. Prior research has emphasized that desert and arid soils, such as those in Egypt, are little-investigated sources of stress-adapted actinomycetes with considerable potential for generating novel antibacterial and therapeutic compounds. Consequently, investigating the Egyptian rhizosphere offers a compelling ecological justification for the isolation of novel bioactive Streptomyces strains^7^.

Previous studies have associated fatty acid derivatives, such as palmitic and stearic acid derivatives, and monoterpene-related compounds such as D-limonene with antioxidant, antimicrobial, or cytotoxic activities. However, in crude extracts, such associations should be considered contextual rather than confirmatory, because the contribution of individual constituents requires isolation, structural confirmation, and direct biological testing^8^.

Advanced computational approaches, including molecular docking, molecular dynamics (MD) simulation, and ADMET prediction, can provide preliminary insight into possible interactions between structurally defined metabolites and selected biological targets. In studies involving crude natural extracts, these approaches should be applied to individual identified or tentatively identified constituents rather than to the extract as a single chemical entity. Accordingly, computational analysis can complement experimental screening by generating hypotheses about possible protein–ligand interactions and by helping prioritize selected metabolites for future fractionation, purification, structural confirmation, and compound-level biological validation. However, such predictions should be interpreted cautiously and should not be considered direct evidence of biological mechanism, antimicrobial or anticancer efficacy, or therapeutic potential without experimental validation^9–11^.

This research investigates a soil-based actinomycete, *Streptomyces paradoxus GH53*, isolated from the Egyptian rhizosphere, for its extract-producing ability and its possible value as a source of metabolites. The strain was identified by morphology and 16 S rRNA sequencing, and fermentation conditions were optimized to increase metabolite yield. A comprehensive analysis of the crude extract was conducted using (GC/MS), (FT-IR), and (UV-Vis) spectroscopy; the extract appeared to contain lipids primarily consisting of saturated fatty acids, esters, terpenoid-related compounds, and related constituents. In addition to biological evaluation through antimicrobial, antioxidant, and cytotoxic assays against WI-38, HePG-2, and MCF-7 cells, the relationship between metabolite structure and biological activity was explored. This involved assessment of the predicted molecular interactions of selected representative GC-MS-identified metabolites against selected antimicrobial and anticancer protein targets (PDB IDs: 3t88, 2xct, 1dgf, 3qfa, 5H38, 4hdq, and 5IRK) using computer-aided drug design techniques such as molecular docking and long-timescale molecular dynamics (MD) simulation.

These computational analyses were therefore used as exploratory tools to support prioritization for future studies, while the biological activities were evaluated experimentally using the crude ethyl acetate extract.

## Experimental section

### Collection of samples and isolation of *Streptomyces sp.*

Samples were collected from rhizosphere soils in El Sharqia Governorate, Egypt (30°68’95.17"N, 31°.82’72.95"E). After drying for 1 h at 60 °C, the samples were serially diluted as described by Hayakawa and Nonomura^12^. The diluted samples were plated on starch nitrate agar medium containing (g/L): starch (20), KNO_3_ (2.0), K_2_HPO_4_ (1.0), CaCO_3_ (3.0), MgSO_4_ (0.5), NaCl (0.5), FeSO_4_ (0.01), and agar (20) at pH 7.8. The medium was sterilized by autoclaving at 121 °C for 15 min, and nystatin (50 U/mL) was added to suppress fungal growth while allowing selective recovery of actinomycetes such as *Streptomyces spp.*

### Preliminary screening for antimicrobial activity

Streptomycetes isolates were primarily screened for their antimicrobial activity against the following target microorganisms, which were adjusted to a 0.5 McFarland standard: Gram-positive bacteria (*Bacillus cereus* ATCC 33018, *Bacillus subtilis* ATCC 6633, and *Staphylococcus aureus* ATCC 25923, *Streptococcus pyogenes* (locally isolated); Gram-negative bacteria (*Escherichia coli* ATCC 8739); yeast (*Candida albicans* ATCC 10231). A lawn culture of the target organisms was made on nutrient agar (Kim et al., 1994) for bacteria. The plates were pre-refrigerated for 2–4 h to allow metabolite diffusion before incubation. Then, the purified isolates of *Streptomycetes* were cultivated for 5 days on starch agar medium. The antimicrobial activity was biologically determined in triplicate by the cylinder diffusion method as follows: a disk of 8.0 mm diameter of each resulting isolate culture was cut and aseptically transferred into holes (8.0 mm diameter) made in the plates seeded with the test organism using a sterile cork borer. The plates were incubated at 30 °C, and the detection of antimicrobial activity was assessed after 24 h by measuring the diameter of the inhibition zone around holes in millimeters^13^.

### Identification of the most potent isolate

The isolate S53, which showed the highest observed antimicrobial activity among the tested isolates, was selected and identified through its morphological, physiological, and biochemical characteristics; additionally, identification was supported by 16 S rRNA gene sequencing, employing the primers F-(5′AGAGTTTGATCMTGGCTCAG-3′) and R-(5′-TACGGYTACCTTGTTACGACTT-3′) as described by Garde’s and Bruns. Following sequencing, the obtained 16 S rRNA sequence was submitted to the GenBank database. Subsequently, it was compared with other 16 S rRNA sequences accessible through the National Center for Biotechnology Information *(*https://www.ncbi.nlm.nih.gov/*)* using the BLAST tool. The sequences of bacterial strains most comparable to the 16 S rRNA gene of our isolate were selected and aligned to construct an appropriate phylogenetic tree, which was generated using MEGA 11 with the bootstrap method (500 replicates)^14^.

### Extraction and purification of the bioactive compounds

*Streptomyces paradoxus GH53* strain was grown under optimized fermentation conditions, and the bioactive compound(s) from culture filtrate were extracted by ethyl acetate, which exhibited the best bioactivity. Then, phase separation was performed using a separatory funnel, the Ethyl Acetate: Filtrate ratio (1:1 v/v) three times, and the upper layer containing bioactive compounds was collected. Finally, the collected upper layer was concentrated in a vacuum rotatory evaporator to separate bioactive compounds from the ethyl acetate^15^. The obtained filtrate was evaporated using rotavapor at 45 °C to give a crude extract, which was stored at 4 °C for bioactivity and chemical profiling of the extract.

### Parameters controlling antimicrobial activity of bioactive metabolites

The fermentation conditions in terms of environmental and nutritional requirements, which affect the antimicrobial activity of bioactive metabolites by *Streptomyces paradoxus* GH53, were optimized by adopting a search technique varying parameters one at a time. Starch-nitrate medium was used as a basal medium for the cultivation of the strain. Each parameter optimized earlier was incorporated in subsequent experiments. This included the effect of the incubation period on the productivity of bioactive metabolites, which were examined daily up to 10 days. The optimum inoculum size was detected using 1, 2, 3, 4, 5, and 6 mL of spore suspension. The optimum temperature for maximum bioactive metabolites yield was measured by incubating the inoculated production medium at 25, 28, 30, and 37 °C. To study the effect of initial pH value, the fermentation medium was adjusted to 5.0, 6.0, 7.0, 8.0, and 9.0 using 1 N HCl or NaOH. For optimizing nutritional requirements, different nitrogen and phosphorus were added to the basal medium instead of starch at an agitation speed of 120 RPM.

### GC-MS analysis

GC-MS analysis of the ethyl acetate extract of *Streptomyces paradoxus* GH53 was performed using a Thermo Scientific ISQ single quadrupole mass spectrometer equipped with an HP-5MS capillary column (30 m × 0.25 mm i.d., 0.25 μm film thickness). The oven temperature was initially maintained at 50 °C for 3 min, then increased at a rate of 10 °C/min to 280 °C, with a final hold for 1 min. Mass spectra were acquired in electron ionization (EI) mode at 70 eV over the range of m/z 40.00–449.97. The chromatographic run covered approximately 32.01 min and included 7943 scans.

Compound annotation was performed by comparing the recorded mass spectra with entries in the NIST mass spectral library, together with retention-time information and relative peak area. Because authentic standards, retention-index validation, and MS/MS fragmentation were not used, the reported GC-MS assignments should be considered tentative rather than definitive structural confirmations.

### FT-IR analysis

Using a KBr pellet method on a Shimadzu IR-Tracer 100 spectrometer, the Fourier Transform Infrared (FT-IR) spectra of the compounds were obtained in a region of 4000–400 cm⁻¹. Approximately 1 mg of each sample was finely ground, mixed with spectroscopic-grade KBr (100 mg), and pressed into transparent pellets under vacuum. A resolution of 4 cm⁻¹ was used to collect the FT-IR spectra, and each spectrum is the result of the average of 32 scans to reduce background noise. The data obtained were analyzed and allowed for a comparative spectral overlay to observe characteristic vibrational modes of the extract from *Streptomyces paradoxus* GH53.

### UV-Visible analysis

The UV-Vis absorption spectra of the extract from *Streptomyces paradoxus GH53* were recorded in methanol solution at room temperature using a double-beam UV-Visible spectrophotometer within the wavelength range 190–550 nm. The solutions were freshly prepared with an approximate concentration of ~ 10 µg/mL. Quartz cuvettes of 1 cm path length were employed, and the baseline was corrected with pure methanol as a reference. The spectra were collected under identical instrumental parameters, including a scanning speed of 200 nm min⁻¹ and a spectral resolution of 1 nm. The UV-Vis absorption spectrum of the crude extract was recorded using a solution prepared at an appropriate concentration expressed in **µg/mL**, rather than molarity, because the extract represents a mixture of compounds rather than a single defined molecular entity.

### Antioxidant activity

#### DPPH free radical scavenging activity

According to Brand Williams et al.^16^, a reaction mixture was prepared by combining 200 µL of 0.1 mM DPPH solution in ethanol with 100 µL of the extract or positive control (ascorbic acid) at concentrations of 0.05 to 0.5 mg/mL. Absorbance was measured at 517 nm. Ethanol was used as the blank, and ascorbic acid served as the reference standard. DPPH radical scavenging activity was calculated using Eq. (1).

1 $$\:DPPH\:Scavening\:activity\:\left(\mathrm{\%}\right)=\left[\left(O.D.\:control-O.D.\:\:sample\right)\div{O.D.}\:\:control\right]\times\:100$$

#### ABTS scavenging activity

The ABTS radical cation scavenging activity of the crude extract and the positive control, ascorbic acid, was evaluated over the concentration range of 0.05–0.5 mg/mL (50–500 µg/mL), according to the method described by Miller and Rice-Evans. The ABTS radical cation scavenging activity was calculated using Eq. (2)^17^.

2 $$\:ABTS\:Scavening\:activity\:\left(\mathrm{\%}\right)=\left[\left(O.D.\:control-O.D.\:sample\right)\div{O.D.}\:control\right]\times\:100$$

### In vitro assessment of antitumor activity

Cell lines from Human lung fibroblast (WI-38), Hepatocellular carcinoma (HEPG-2), and Mammary gland breast cancer (MCF-7) were obtained from ATCC via Holding Company for Biological Products and Vaccines (VACSERA), Cairo, Egypt, for the purpose of evaluating their cytotoxicity and viability. 10% fetal bovine serum (FBS), 1% glutamine, and 1% penicillin/streptomycin were added to RPMI 1640 media in order to nourish the cells. The cells were kept in a humidified incubator with 5% CO_2_ at 37 °C. The Methyl Thiazolyl Tetrazolium (MTT) viability test was used to assess the cytotoxicity of the extract^18^. Every test was run three times for 48 h, and the average of the results was calculated. The following formula was used to determine cell viability as the following Eq. (3):

3 $$\:Cell\:inhibition\:\left(\mathrm{\%}\right)=\left[\left(O.D.\:control-O.D.\:sample\right)\div{O.D.}\:control\right]\times\:100$$

### ADMET analysis

ADMET prediction was performed only for selected representative metabolites tentatively identified by GC-MS, not for the crude ethyl acetate extract as a whole. The selected metabolites, including D-limonene, n-hexadecanoic acid, palmitic anhydride, L-(+)-ascorbic acid 2,6-dihexadecanoate, and docosanoic acid 1,2,3-propanetriyl ester, were treated as individual, structurally defined ligands for computational evaluation. This approach was adopted because standard ADMET descriptors, such as molecular weight, logP, hydrogen-bond donors and acceptors, topological polar surface area, rotatable bonds, solubility, gastrointestinal absorption, blood–brain barrier permeability, cytochrome P450 interaction, and predicted toxicity, are meaningful only for single defined chemical structures and cannot be scientifically assigned to a crude extract or metabolite mixture.

In the initial analysis, an unrealistically high molecular weight value of 3695 Da and logP value of 54.4 were obtained. Therefore, these descriptors were considered invalid for standard ADMET interpretation and were excluded from the final analysis. The ADMET descriptors were subsequently recalculated and re-reported for each selected GC-MS-identified metabolite individually. The corrected compound-level values are presented in Tables 1, 2, 3 and were used for the revised interpretation.

**Table 1:** Different characteristics of the streptomycete isolate (S53).

Morphological and cultural characteristics																
Spore chain morphology				Spore surface ornamentation				Color of spore mass		Pigmentation of substrate mycelium				Diffusible Pigment		
Straight/Flexuous > 40				Smooth				Gray		grayish white				-ve		
Physiological and biochemical characteristics																
Melanin pigment Production					Degradation activities							Nitrate reduction			H_2_S production	
peptone iron		Tyrosine			xanthine		elastin		arbutin			**+ve**			**+ve**	
**+ve**		**+ve**			**-ve**		**-ve**		**+ve**							
Utilization of sugars																
D-fructose	Sucrose		Rhamnose			D-mannitol		D-xylose	Raffinose		I-inositol		Galactose			L-arabinose
**-ve**	**-ve**		**-ve**			**+ve**		**+ve**	**+ve**		**-ve**		**+ve**			**+ve**

**Table 2:** Major GC-MS identified components of the extract from *Streptomyces paradoxus* GH53.

Peak	RT (min)	Compound (library match)	Class/description	Molecular formula	MW (Da)	Area (%)
1	8.71		*Monocyclic monoterpene*	C₁₀H₁₆	136	8.05
2	18.56		*Straight-chain alkane*	C₁₆H₃₄	226	1.17
3	18.56		*Branched isoparaffin (C₂₀ alkane)*	C₂₀H₄₂	282	1.17
4	18.56		*Branched C₁₈ alkane*	C₁₈H₃₈	254	1.17
5	~ 23–26		*Branched C₁₆ alkane (tentative)*	C₂₀H₄₂†	282†	~ 3–5
6	28.51		*Saturated fatty acid*	C₁₆H₃₂O₂	256	28.15
7	28.51		*Fatty acid anhydride (dimer of C16)*	C₃₂H₆₂O₃	494	28.15‡
8	28.51		*Ascorbyl palmitate diester*	C₃₈H₆₈O₈	652	28.15‡
9	29.44		*Glycerol triester of behenic acid*	C₆₉H₁₃₄O₆	1058	1.55

Compound identities are tentative and were assigned primarily by NIST library matching without confirmation by authentic standards or MS/MS fragmentation analysis.

**Table 3:** Physicochemical and predicted ADMET-relevant descriptors of selected GC-MS-identified metabolites from the ethyl acetate extract of *Streptomyces paradoxus GH53*.

Metabolite	MW (Da)	logP	HBD	HBA	Rotatable bonds	TPSA (Å²)
D-limonene	136.23	4.23	0	0	0	0.0
n-Hexadecanoic acid	256.42	7.03	1	2	14	37.3
Palmitic anhydride	494.82	12.75	0	3	29	52.6
L-(+)-Ascorbic acid 2,6-dihexadecanoate	764.06	13.77	2	8	31	146.0
Docosanoic acid, 1,2,3-propanetriyl ester	1061.76	20.68	0	8	63	104.6

(D-limonene, n-Hexadecanoic acid, Palmitic anhydride, L-(+)-Ascorbic acid 2,6-dihexadecanoate and Docosanoic acid, 1,2,3-propanetriyl ester were selected as individual metabolites identified by GC-MS and selected for the in silico study, not for the crude extract as a single molecular entity. These data are presented to compare the molecular size, lipophilicity, polarity, flexibility, and predicted drug-likeness-related properties of representative constituents detected in the extract.

Note: The previously obtained MW 3695 Da and logP 54.4 values were not retained because they resulted from an invalid extract-level or mixture-level input. The values reported in this table correspond only to individual GC-MS-selected metabolites and should be interpreted as preliminary predicted descriptors for defined compounds, not for the crude extract.

The crude ethyl acetate extract of *Streptomyces paradoxus* GH53 should therefore be regarded as a complex mixture of metabolites, whereas the ADMET results represent preliminary compound-level predictions for selected individual constituents. The experimentally observed antioxidant, antimicrobial, and cytotoxic activities were evaluated using the whole crude extract and may reflect the combined, additive, or synergistic effects of multiple constituents. In contrast, the ADMET analysis was intended only to provide an initial pharmacokinetic and toxicity-related screening of representative metabolites that may contribute to the observed extract-level bioactivities.

Because the GC-MS assignments were based primarily on library matching and were not confirmed by authentic standards, retention-index validation, or MS/MS fragmentation, the ADMET results should be interpreted cautiously as exploratory predictions. These data are useful for prioritizing metabolites for future isolation, purification, structural confirmation, and compound-level biological testing, but they do not establish drug-likeness, safety, or therapeutic suitability of the crude extract. In particular, highly lipophilic and high-molecular-weight metabolites, such as long-chain fatty acid derivatives and glyceride-like constituents, may show limited aqueous solubility, reduced predicted oral absorption, extensive plasma protein binding, and possible toxicity liabilities. Therefore, further experimental pharmacological and toxicological validation is required before any pharmaceutical relevance can be confirmed.

### Docking analysis and simulation

The selected GC-MS-identified metabolites were docked as individual ligands rather than treating the crude extract as a single chemical entity. Ligand preparation and docking were performed using standard small-molecule workflows in MOE and Discovery Studio Client version 4.2, with confirmatory scoring using AutoDock Vina after geometry preparation^19^. Molecular dynamics simulations of the resulting protein–ligand complexes were then carried out in GROMACS^20^ in explicit water at 300 K using standard protein and ligand force-field parameters.

The selected PDB targets were chosen as representative proteins related to the experimentally evaluated antimicrobial, antioxidant, and antitumor activities. For antimicrobial-related analysis, the selected targets included the twinned 3.35 Å structure of *S. aureus* gyrase complex with ciprofloxacin and DNA, PDB ID: 2XCT^21^ and the Crystal structure of *Escherichia coli* MenB in complex with substrate analogue, OSB-NCoA (PDBID:3t88)^24^, For antioxidant-related activity The HUMAN ERYTHROCYTE CATALASE(PDBID: 1dgf)^22^ and PDBID:3qfa(Crystal structure of the human thioredoxin reductase-thioredoxin complex)^23^, For antitumor-related analysis Heart of Glass (HEG1) cytoplasmic tail and Rap1 GTPase- and KRIT1’s Ternary Complex crystallographic structure PDBID: (4hdq)^24^ and Structural analysis of KSHV thymidylate synthase PDBID:5H38^25^, The Structure of Mefenamic Acid Bound to Human Cyclooxygenase-2 (PDBID:5IRK)^26^.Ten docking runs were performed for each ligand–target pair. The ligands used for docking were selected individual GC-MS-identified metabolites, namely D-limonene, n-hexadecanoic acid, palmitic anhydride, L-(+)-ascorbic acid 2,6-dihexadecanoate, and docosanoic acid 1,2,3-propanetriyl ester. The docking and MD results are reported in Tables 4, 5 and 6. Because no direct experimental metabolite–target validation was performed, the docking and MD analyses were interpreted as preliminary, hypothesis-generating computational evidence rather than proof of mechanism or biological efficacy^27^.

**Table 4:** Molecular docking parameters of selected GC-MS-identified metabolites from the *Streptomyces paradoxus* GH53 extract against the antimicrobial targets 3t88 and 2xct.

Compound	Binding Energy (B. E)		Binding distance	Inhibitory constant, Ki (µM)	Binding amino acids	vdW + H bond +desolv Energy	Electrostatic energy	Total Internal, Unbound Energy	ΔG	RMSD
PDBID:3t88 (Antimicrobial)										
Representative GC-MS-selected metabolite from the exploratory ligand set		−11.43	2.22–3.40	5.52	ASP 17, GLU 28, ILE 34 ALA 35, HIS 16, CYS 18, LYS 36, TRP 15, LYS 29, GLY 33, ILE 69, TYR 27 VAL 71, SER 30	−16.2	−21.2	−12.42	−22.02	0.92
*PDBID:2xct (Antimicrobial)*										
Representative GC-MS-selected metabolite from the exploratory ligand set	−9.43		2.489, 2.647, 2.786, 2.812, 3.114, 3.321, 3.323 3.411	6.22	SER-1084, ARG-458 GLY-459, ARG-458 GLU-477	−10.53	−18.43	−13.83	−20.54	0.95

Selected GC-MS-identified metabolite (D-limonene, n-Hexadecanoic acid, Palmitic anhydride, L-(+)-Ascorbic acid 2,6-dihexadecanoate and Docosanoic acid, 1,2,3-propanetriyl ester).Binding energy, contact distances, estimated inhibitory constant, interacting amino acid residues, van der Waals/hydrogen-bond/desolvation contributions, electrostatic energy, total internal energy, ΔG, and RMSD are reported for each compound–target complex. The results are intended to provide a compound-level mechanistic interpretation and should not be interpreted as direct docking of the crude extract as a single ligand.

**Table 5:** Molecular docking parameters of selected GC-MS-identified metabolites from the *Streptomyces paradoxus* GH53 extract against the antioxidant-related targets 1dgf and 3qfa.

Compound	Binding Energy (B. E)	Binding distance	Inhibitory constant, Ki (µM)	Binding amino acids	vdW + H bond +desolv Energy	Electrostatic energy	Total Internal, Unbound Energy	ΔG	RMSD
*PDBID:1dgf (Antioxidant)*									
Representative GC-MS-selected metabolite from the exploratory ligand set	−10.53	2.7–3.2	4.93	GLU 453, GLU 454, LYS 457, ASP 488, ARG 456, ARG 458, ASN 452, GLN 455, HIS 492, SER 491	−12.93	−22.73	−11.83	−26.38	0.91
*PDBID:3qfa(Antioxidant)*									
Representative GC-MS-selected metabolite from the exploratory ligand set	−12.83	2.76 2.91 2.77 3.12	3.84	Glu136, Glu137, Asp176 Lys179, Tyr180, Lys183	−13.83	−25.73	−14.83	−29.7	0.90

Selected GC-MS-identified metabolite (D-limonene, n-Hexadecanoic acid, Palmitic anhydride, L-(+)-Ascorbic acid 2,6-dihexadecanoate and Docosanoic acid, 1,2,3-propanetriyl ester).Binding energy, contact distances, estimated inhibitory constant, interacting amino acid residues, van der Waals/hydrogen-bond/desolvation contributions, electrostatic energy, total internal energy, ΔG, and RMSD are reported for each compound–target complex. The results are intended to provide a compound-level mechanistic interpretation and should not be interpreted as direct docking of the crude extract as a single ligand.

**Table 6:** Molecular docking parameters of selected GC-MS-identified metabolites from the *Streptomyces paradoxus* GH53 extract against the anticancer-related targets 5H38, 4hdq, and 5IRK.

Compound	Binding Energy (B. E)	Binding distance	Inhibitory constant, Ki (uM)	Binding amino acids	vdW + H bond +desolv Energy	Electrostatic energy	Total Internal, Unbound Energy	ΔG	RMSD
*PDBID:5H38(antitumor)HepG2*									
Representative GC-MS-selected metabolite from the exploratory ligand set	**−10.32**	2.10–2.40	**3.04**	**GLU453**,** ARG456**,** ASN | 452**,** THR128**,** ASP318** **GLY129**,** ARG106**	**−12.43**	**−23.83**	**−10.32**	**−21.82**	**0.93**
***PDBID:4hdq (Antitumor)(MCF-7)***									
Representative GC-MS-selected metabolite from the exploratory ligand set	**−8.43**	**2.52**,** 2.57** **2.62**,** 2.68** **2.79**,** 2.89** **2.93**,**2.94**	**6.32**	**VAL B29**,** ASP B33**,** LYS B16**,** VAL B29**,** ASP B38** **GLN B63**,** GLU B37** **SER B17**	**−9.33**	**−21.42**	**−11.54**	**−22.64**	**0.99**
***PDBID:5IRK(Antitumor)(WI-38)***									
Representative GC-MS-selected metabolite from the exploratory ligand set	−15.32	2.39, 2.69, 2.77 2.82, 3.20, 3.20 3.18, 3.24, 3.27, 3.32 Å	4.645	TYR385B, SER530B SER530A, TYR385A VAL349A, SER530A TYR385B, SER530B VAL523B, VAL349	−11.32	−23.64	−10.43	−21.53	0.94

Selected GC-MS-identified metabolite (D-limonene, n-Hexadecanoic acid, Palmitic anhydride, L-(+)-Ascorbic acid 2,6-dihexadecanoate and Docosanoic acid, 1,2,3-propanetriyl ester).Binding energy, contact distances, estimated inhibitory constant, interacting amino acid residues, van der Waals/hydrogen-bond/desolvation contributions, electrostatic energy, total internal energy, ΔG, and RMSD are reported for each compound–target complex. The results are intended to provide a compound-level mechanistic interpretation and should not be interpreted as direct docking of the crude extract as a single ligand.

### Statistical analysis

All experiments were performed in triplicate, and the results are expressed as mean ± standard deviation (SD). Statistical analyses were carried out using GraphPad Prism version 10. For optimization experiments evaluating the effects of incubation time, inoculum size, temperature, pH, nitrogen sources, and phosphate sources on antimicrobial activity, data were analyzed using one-way analysis of variance (ANOVA). For antioxidant assays, comparisons between the crude extract and the reference standard were performed using an unpaired test with Welch’s correction, for DPPH and ABTS radical scavenging activities were calculated using Fit spline/LOWESS analysis. Statistical significance was considered at *p* < 0.05, and error bars in the figures represent the SD of triplicate measurements.

## Results and discussion

### Isolation, screening, and identification of Streptomyces

**Fig. 1:**
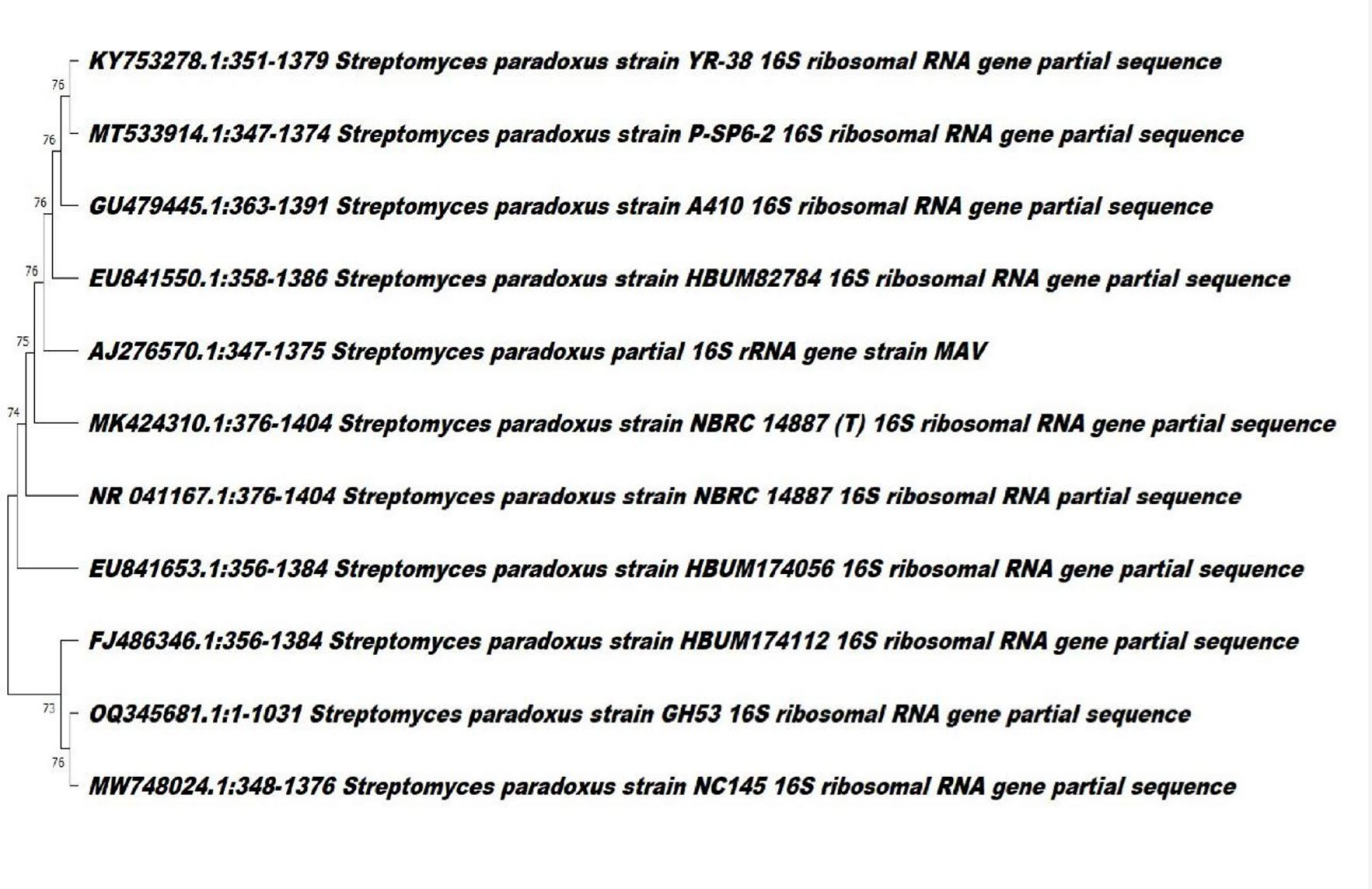
Phylogenetic tree based on the partial 16 S rRNA gene sequence of the local isolate *Streptomyces paradoxus* GH53 and closely related sequences available in the GenBank database.

Seventy-five *Streptomyces* isolates were obtained from the El Sharqia Governorate and given the names S1 to S75 due to their distinct morphological and cultural characteristics, which are usually round, convex-shaped colonies and their rooting growth into the medium. Among these isolates, S53 showed higher antimicrobial potency compared to other species. Isolate S53 showed straight, flexuous spore chain morphology with smooth spore surface ornamentation. The color of the spore mass was gray, and the substrate mycelium pigmentation was grayish white. The isolate showed no ability for diffusible pigment. But it can produce melanoid pigment, and arbutin is degraded actively, reducing nitrate and producing hydrogen sulfide. Utilization of sugars has been performed by using D-glucose as a positive control; it can utilize D-mannitol, D-xylose, Raffinose, galactose, and arabinose **(**Table 1). The identification of *Streptomyces sp.* was confirmed through molecular analysis of the 16 S rRNA partial sequence using its genomic DNA as a template for PCR. The PCR amplicon was subsequently purified, sequenced, and BLAST searched against a non-redundant database. As a result, it was identified as *Streptomyces paradoxus* GH53 with accession number OQ345681.1. The phylogenetic tree of the local isolate *Streptomyces paradoxus* GH53’s partial 16 S rRNA sequence in relation to closely related sequences from GenBank databases were displayed in Fig. 1.

### Parameters controlling the productivity of bioactive metabolites

#### Effect of incubation period, inoculum size on bioactive metabolite production

**Fig. 2:**
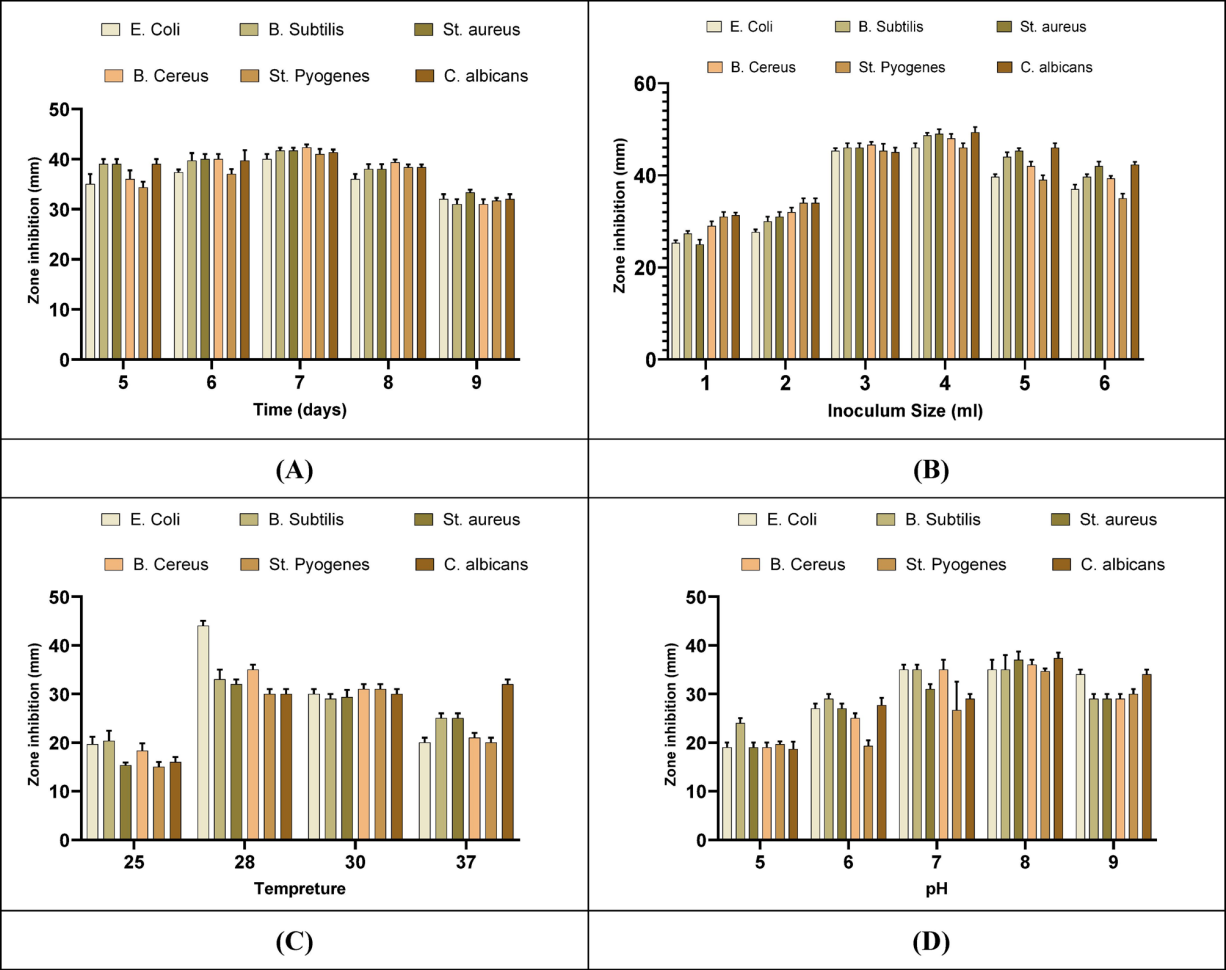
Effect of different fermentation parameters on the antimicrobial activity of bioactive metabolites produced by *Streptomyces paradoxus* GH53: (A) incubation time, (B) inoculum size, (C) temperature, and (D) pH. Data are presented as mean ± SD. One-way ANOVA was used for data analysis (*n* = 3). Statistical significance was considered at *p* < 0.05.

The antimicrobial activity of metabolites produced by the selected isolate was monitored over a period of 9 days. The bioactive metabolite reached a maximum for all tested organisms on the 7th day, then decreased on the 9th day, as shown in Fig. 2A. Statistical analysis revealed that the activity observed on day 7 was significantly higher than that recorded on days 5 and 9 (*p* < 0.05). Thereafter, a significant decline in activity was observed by day 9. By increasing the inoculum size, the antimicrobial activity of the bioactive metabolites increased until it reached 4 mL for all the tested organisms, as seen in Fig. 2B, then decreased by increasing it. The activity at 4 mL was significantly higher than that obtained at lower or higher inoculum sizes (*p* < 0.05). Further increases in inoculum size resulted in a significant reduction in antimicrobial activity.

#### Effect of incubation temperature and PH on bioactive metabolite production

The effect of incubation temperature on the antimicrobial activity of bioactive metabolites was studied for all the selected organisms. 28˚C was observed to be the optimum temperature for bioactive metabolite production. By increasing and decreasing the temperature (28˚C), production was decreased as noticed in Fig. 2C. The activity at this temperature was significantly higher than that observed at 25, 30, and 37 °C (*p* < 0.05), confirming 28 °C as the optimum temperature for metabolite production. The effect of initial pH on the antimicrobial activity of the bioactive metabolite was studied. It was maximum for all tested organisms at pH 7 (Fig. 2D), and this activity was significantly higher than that observed at pH 5 and pH 9 (*p* < 0.05). A marked reduction in activity was observed under more acidic or alkaline conditions.

#### Effect of nitrogen sources on bioactive metabolite production

**Fig. 3:**
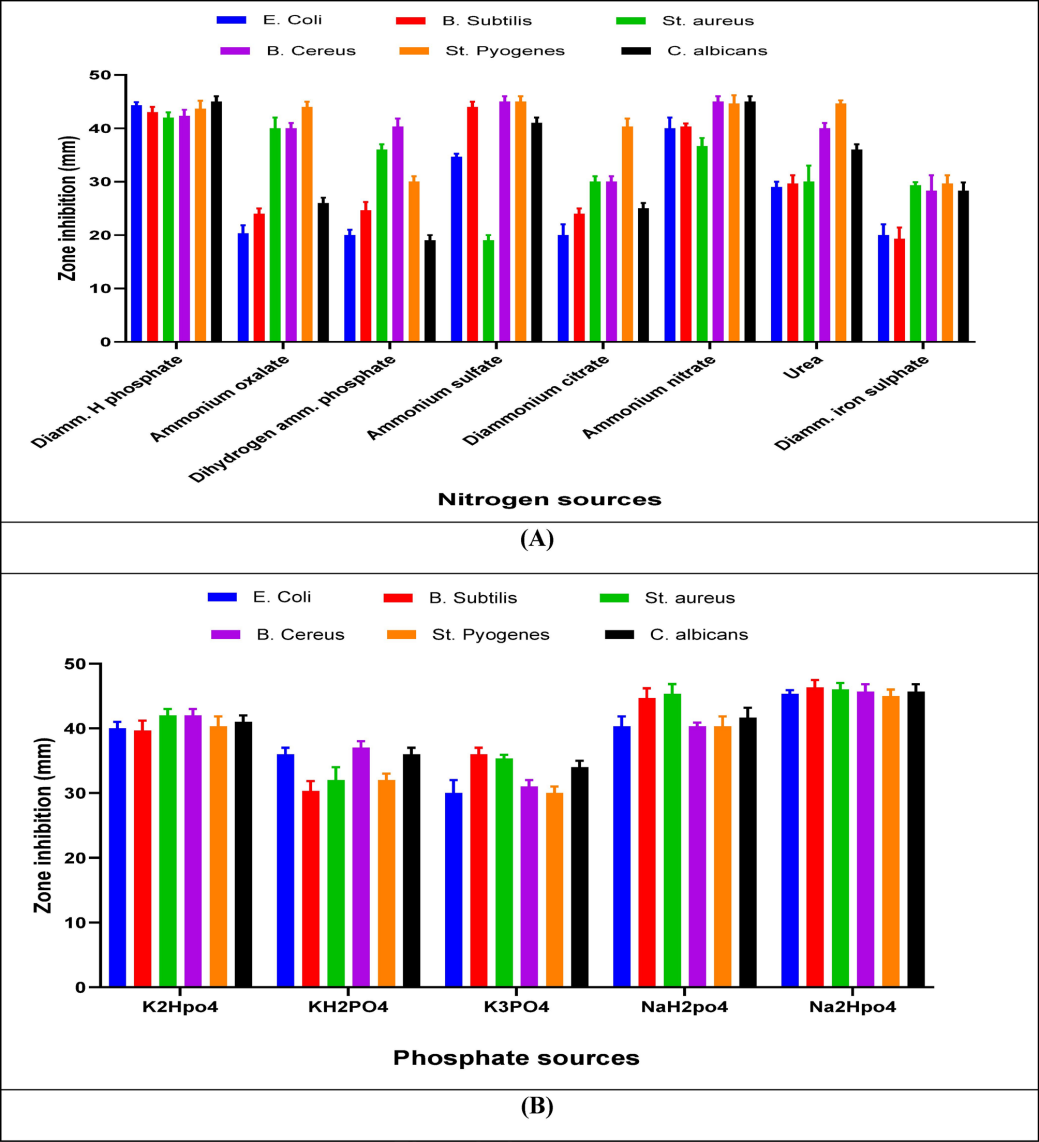
Effect of different nutrient sources on the antimicrobial activity of bioactive metabolites produced by *Streptomyces paradoxus* GH53: (A) nitrogen sources and (B) phosphate sources. Data are presented as mean ± SD. One-way ANOVA was used for data analysis (*n* = 3). Statistical significance was considered at *p* < 0.05.

It was clear from the results that the growth of the isolate was greatly influenced by the nature and type of nitrogen source supplemented in the medium. In comparison with the inorganic nitrogen sources, organic nitrogen sources induced relatively higher biomass yield as well as bioactive metabolite production. In this study, potassium nitrate induced the production of the bioactive metabolite when compared with the other sources as noticed in Fig. 3A, showing a statistically significant increase compared with the other nitrogen sources tested (*p* < 0.05).

#### Effect of phosphate sources on bioactive metabolite production

By studying the effect of different phosphate sources and comparing them it is obvious that Na_2_HPO_4_ induced the antimicrobial activity of extract followed by NaH_2_PO_4_ and K_2_HPO_4_ respectively as seen Fig. 3B. Statistical analysis demonstrated that Na₂HPO₄ produced significantly greater activity than the other phosphate sources tested (*p* < 0.05).

### GC-MASS

**Fig. 4:**
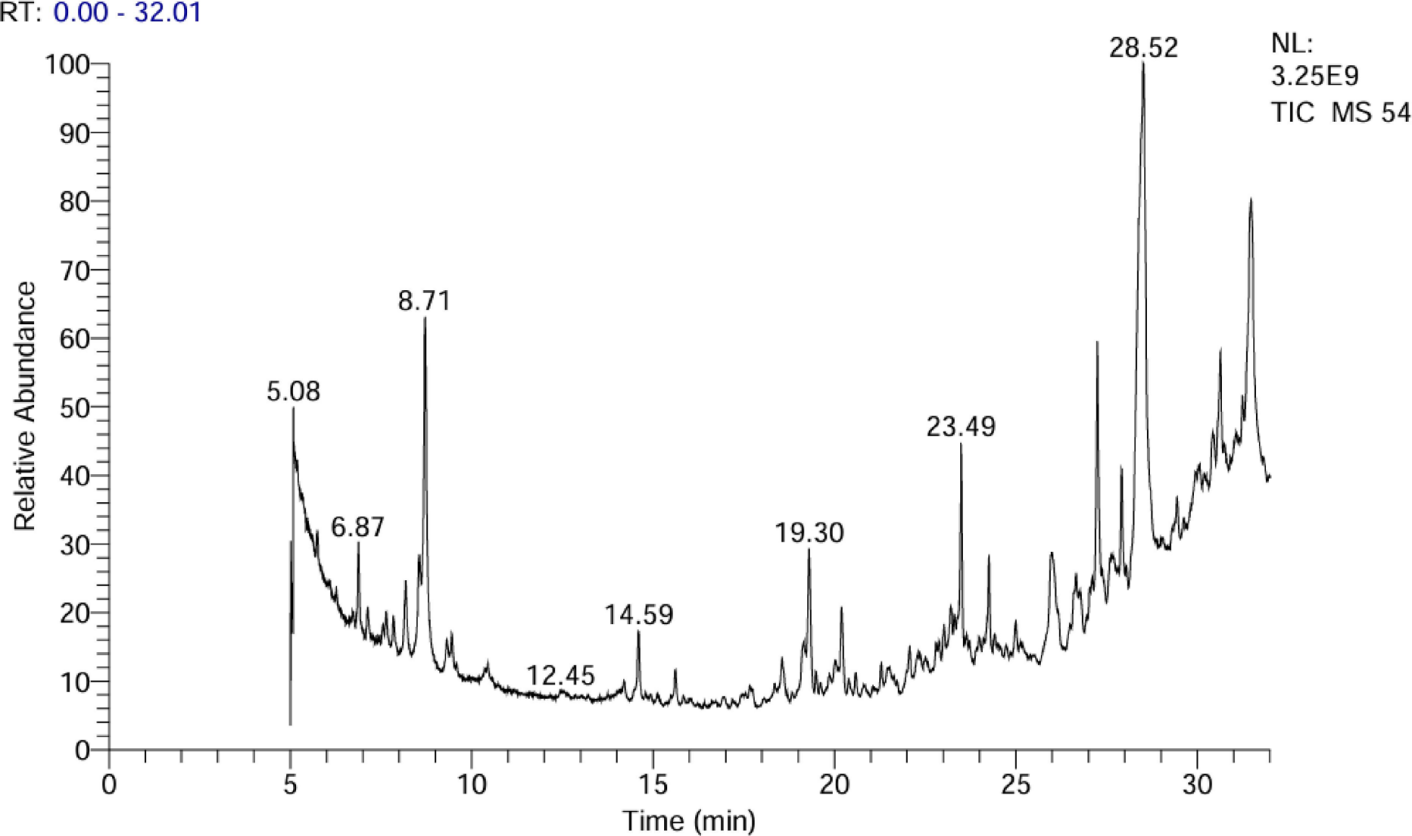
GC-MS chromatogram of the ethyl acetate extract of *Streptomyces paradoxus* GH53.

The GC-MS chromatogram of the ethyl acetate extract of *Streptomyces paradoxus* GH53 showed a complex metabolic profile, with several peaks distributed between approximately 6.8 and 29.4 min (Fig. 4; Table 2). Compound annotation was performed by comparison of the recorded mass spectra with entries in the NIST library, together with retention-time information and relative peak area. Because no authentic standards, retention-index validation, or MS/MS fragmentation analysis were used, the detected compounds should be regarded as tentatively identified constituents rather than definitively confirmed structures.

The chromatographic profile indicated the presence of several metabolite classes, mainly fatty acids, fatty acid derivatives, hydrocarbons, and terpenoid-related constituents. The most prominent signal appeared at RT 28.51 min and represented 28.15% of the total peak area. This peak was assigned mainly to n-hexadecanoic acid, with possible contribution from related lipid-derived constituents. A second notable peak at RT 8.71 min, representing 8.05% of the total peak area, was assigned to D-limonene, suggesting the presence of a monoterpene-related constituent. Additional peaks at RT 18.56, 23.49, 25.98, and 29.44 min were associated with long-chain hydrocarbons, branched alkanes, fatty acid esters, and higher-molecular-weight lipid derivatives, including compounds such as palmitic anhydride, L-(+)-ascorbic acid 2,6-dihexadecanoate, and docosanoic acid 1,2,3-propanetriyl ester.

Overall, the GC-MS profile suggests that the GH53 extract is a lipid-rich and chemically complex crude mixture. These results provide a preliminary chemical basis for interpreting the observed extract-level bioactivities and for selecting representative metabolites for computational screening. Accordingly, D-limonene was selected as a representative monoterpene-related constituent, while n-hexadecanoic acid, palmitic anhydride, L-(+)-ascorbic acid 2,6-dihexadecanoate, and docosanoic acid 1,2,3-propanetriyl ester were selected as representative fatty acid or lipid-derived constituents for docking, molecular dynamics, and ADMET analyses. This selection was based on relative abundance, chemical relevance, and representation of the major metabolite classes detected in the extract.

### FT-IR analysis

**Fig. 5:**
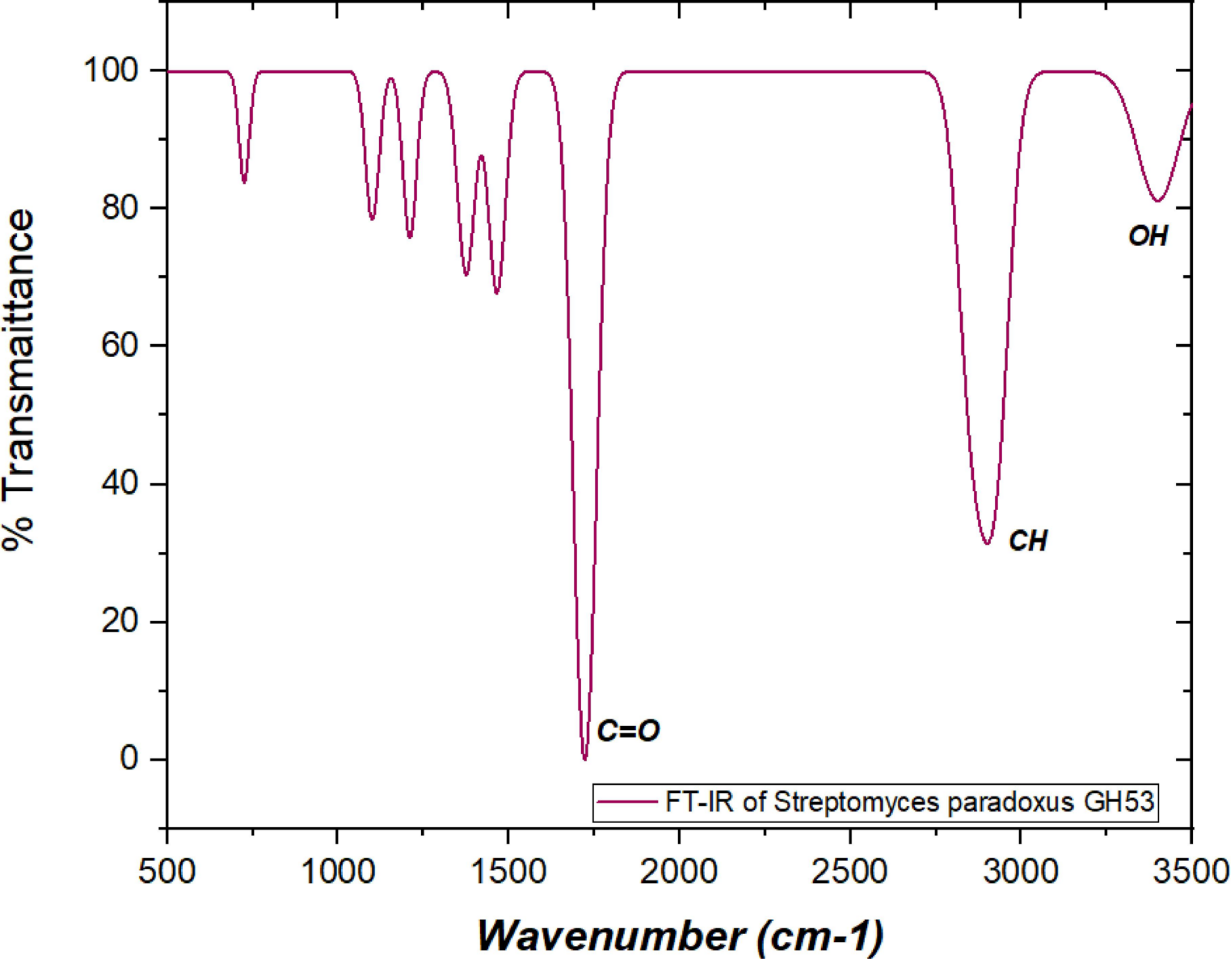
FT-IR spectrum of the ethyl acetate extract isolated from *Streptomyces paradoxus* GH53.

The FT-IR spectra for GH53 samples of *Streptomyces paradoxus* suggest the presence of fatty acids, esters, and/or hydrocarbon chains, in a manner consistent with the GC/MS profile shown in Fig. 5. The presence of saturated fatty acids such as palmitic and stearic acids may be reflected in the strong band around 1700 cm⁻¹, which can be assigned to carbonyl (C = O) stretching. A well-defined band between approximately 2920 and 2850 cm⁻¹, attributed to asymmetric and symmetric stretching of aliphatic –CH₂/–CH₃ groups, is also consistent with long-chain hydrocarbons and branched alkanes identified by GC/MS. The broad signal near 3300 cm⁻¹, assigned to O–H stretching, may reflect hydrogen-bonded hydroxyl groups of carboxylic acids as well as residual moisture. Several peaks in the fingerprint region (1000–1500 cm⁻¹) can be assigned to C–O, C–C, and C–H bending modes associated with fatty acids, esters, glycerides, and related constituents. Taken together, the FT-IR data support, but do not by themselves prove, the presence of lipid-rich constituents containing carboxylic acid and hydrocarbon functional groups, in agreement with the GC/MS analysis.

### UV-Visible analysis

**Fig. 6:**
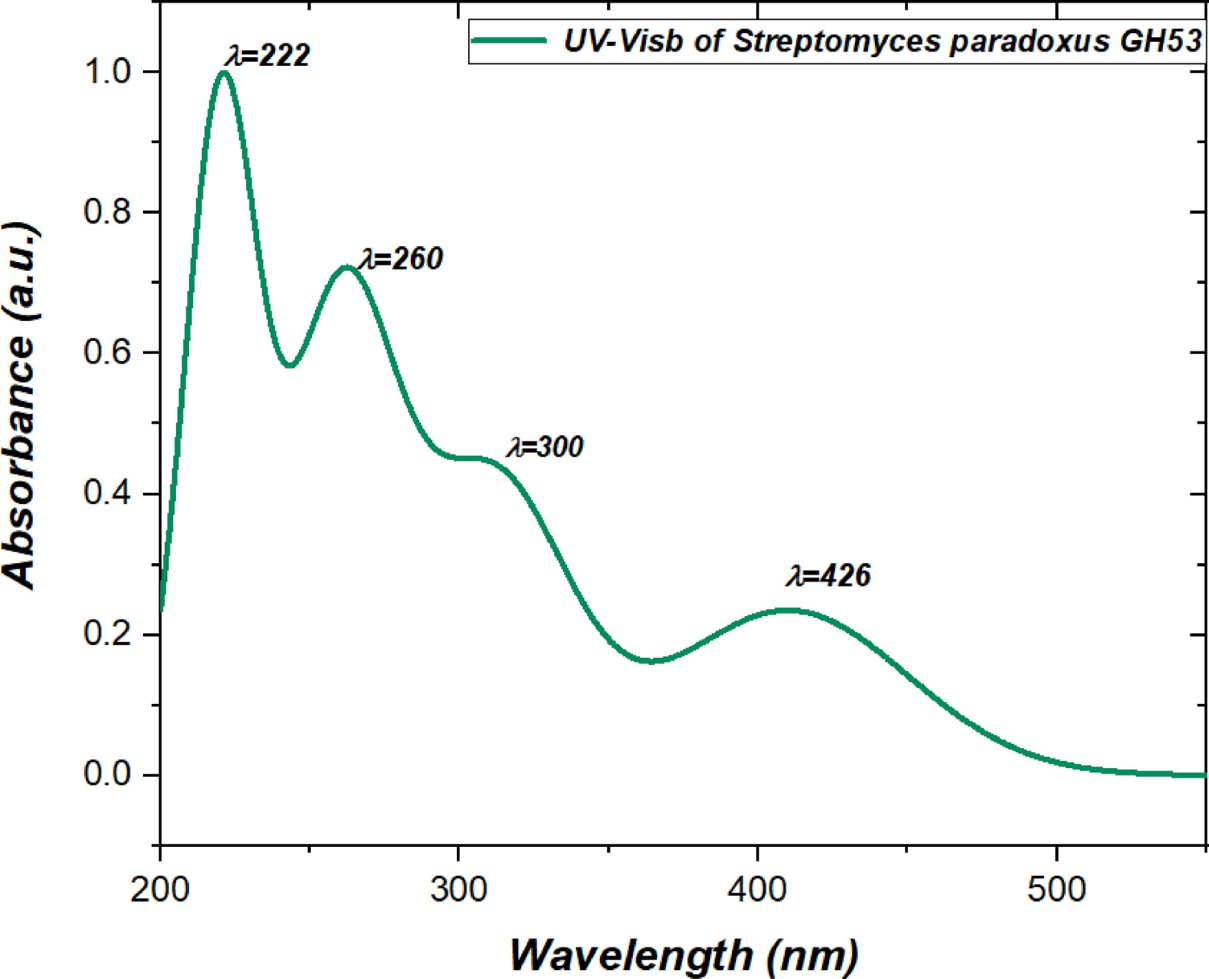
UV-visible absorption spectrum of the ethyl acetate extract isolated from *Streptomyces paradoxus* GH53.

The UV-Vis spectral fingerprint of the crude extract of *Streptomyces paradoxus* GH53 showed bands at approximately 222, 260, 300, and 426 nm suggest absorbing chromophores associated with π→π* and n→π* transitions in unsaturated, carbonyl-containing, and pigment-related constituents. These features are consistent with the occurrence of terpenoid-related compounds, fatty acids, fatty acid esters, and other oxygenated metabolites suggested by the GC-MS profile and supported by the FT-IR data. Thus, the main significance of Fig. 6 is to provide complementary spectroscopic support for the chemical classes that may be present in the extract and to indicate that the pigment fraction contains electronically active components; however, UV-Vis data alone are not sufficient for unambiguous structural assignment of individual metabolites. Accordingly, Fig. 6 should be interpreted as a supporting characterization figure that strengthens the internal consistency of the analytical dataset rather than as an independent source of definitive molecular identification (Fig. 6).

### ADMET analysis of selected GC-MS-identified metabolites

**Fig. 7:**
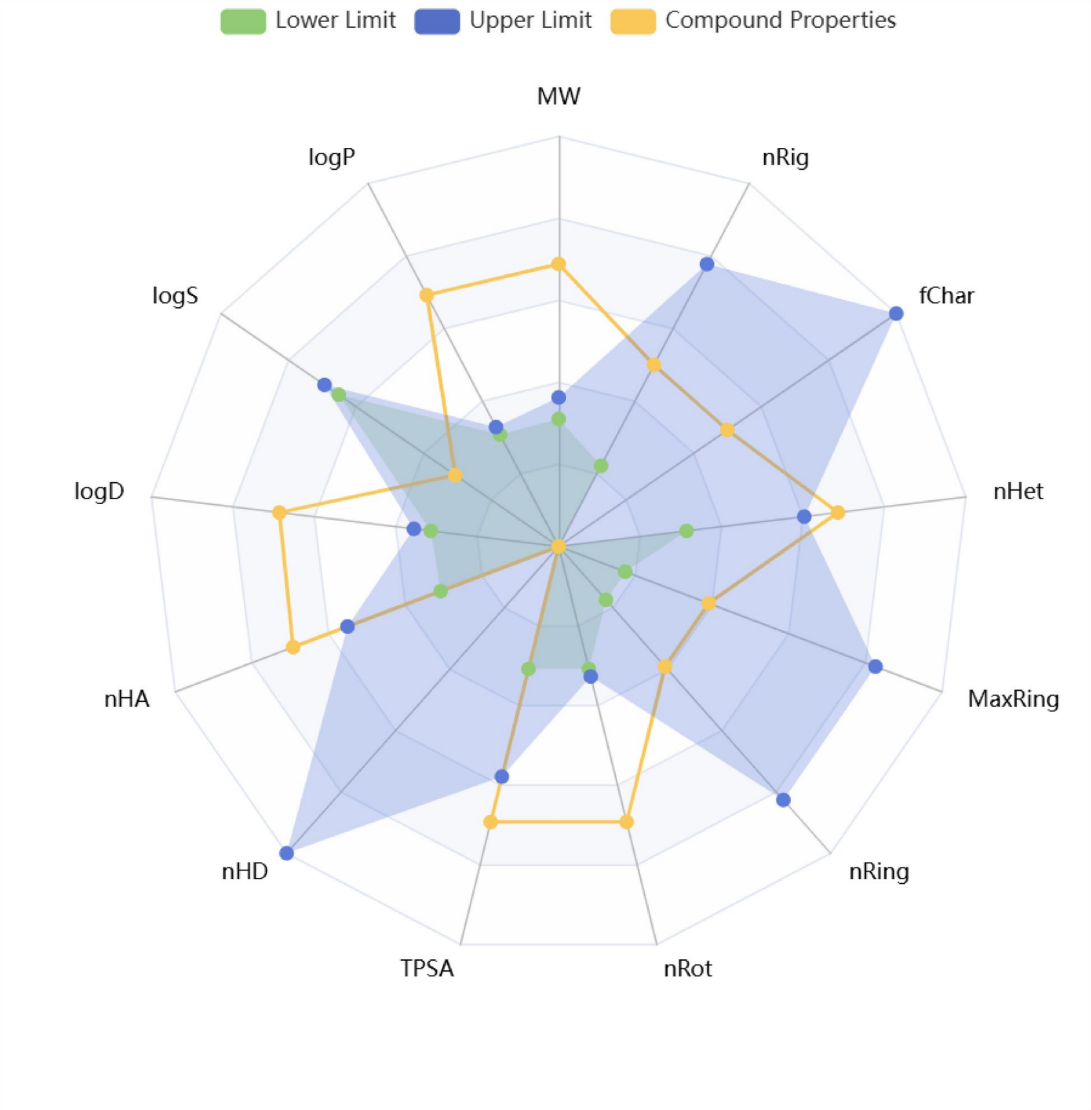
Radar chart comparing the physicochemical and ADMET-related parameters of selected GC-MS-identified metabolites from the *Streptomyces paradoxus* GH53 extract against standard acceptable lower and upper limits.

ADMET prediction was restricted to selected GC-MS-identified metabolites treated individually (Fig. 7; Table 3). The resulting descriptor space was broad: smaller constituents such as D-limonene lie closer to conventional small-molecule space, whereas long-chain esterified lipids and glyceride-like constituents show substantially higher molecular weight, stronger lipophilicity, lower expected aqueous solubility, and weaker conformity with common drug-likeness guidelines. Accordingly, the ADMET output should be interpreted only as preliminary compound-level screening information and not as evidence that the crude extract itself is drug-like. Overall, the predictions suggest that the more hydrophobic, higher-mass constituents are more likely to show limited oral absorption, extensive plasma protein binding, and broader pharmacokinetic liabilities, whereas the smaller metabolites may be more tractable from a physicochemical standpoint. These predictions are useful for prioritizing future fractionation and compound-level validation, but they do not establish therapeutic suitability without full structure confirmation and dedicated pharmacology and toxicology studies^27,28^.

### Biological analysis

The biological assays were performed on a crude extract rather than on isolated compounds and, no direct compound-level biological activity can be concluded for the individual GC-MS-identified constituents without further isolation and validation. So, the observed activities should be interpreted as preliminary extract-level effects and not as definitive evidence of the potency of any specific metabolite.

#### Antioxidant activity

**Fig. 8:**
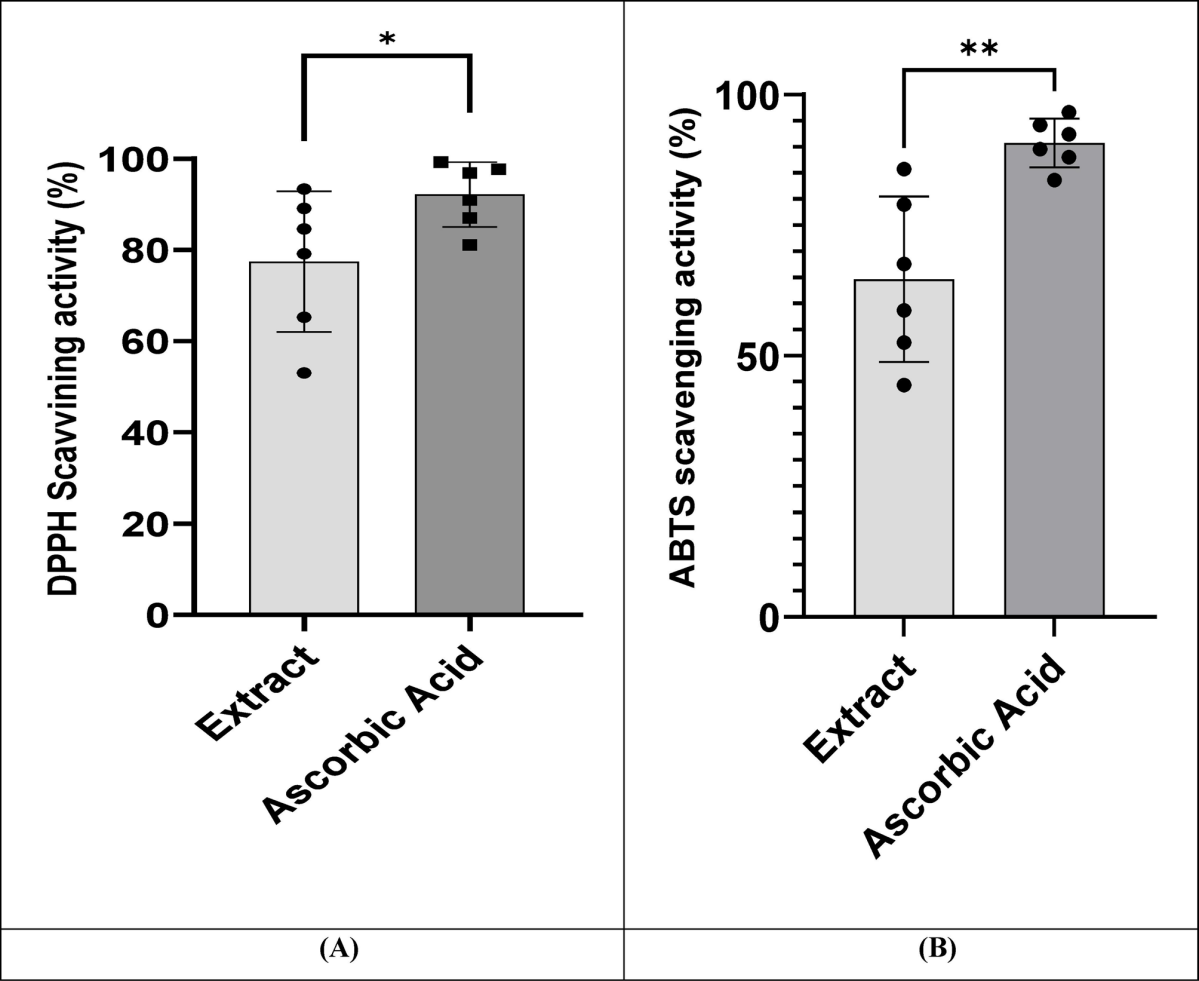
(A) DPPH radical scavenging activity and (B) ABTS radical scavenging activity of the bioactive extract produced by *Streptomyces paradoxus* GH53. Data are presented as mean ± SD. An unpaired test with Welch’s correction was used for data analysis (*n* = 3). Statistical significance was considered at *p < 0.05 and **p < 0.01. IC₅₀ values were calculated using Fit spline/LOWESS analysis.

The DPPH test was used to evaluate the extract’s capacity to scavenge free radicals in relation to standard components (ascorbic acid), as illustrated in Fig. 8A. Increasing the dosages from 0.05 to 0.5 mg/ml significantly enhanced the DPPH radical scavenging capacity, according to the results given. It was increased from 53.02 ± 0.46% to 93.34 ± 0.63%. While the ascorbic acid scavenging percentage rose from 81.07 ± 0.81% to 99.24 ± 0.35%. By using ABTS for the detection of antioxidant activity, by increasing concentrations from 0.05 to 0.5 mg/mL, the antioxidant activity increases from 44.31 ± 0.60% to 85.70 ± 0.95%. Therefore, Ascorbic acid scavenging percentage increases from 80.22 ± 0.25% to 96.58 ± 0.33% as shown in Figure (8 B). The IC_50_ of the extract was 0.175 ± 0.013 and 0.097 ± 0.006 mg/mL for DPPH and ABTS, respectively.

#### Antitumor activity against different cell lines

Antitumor activity was evaluated by the MTT assay against WI-38, HePG-2, and MCF-7 cells. The extract produced a concentration-dependent reduction in cell viability, with IC50 values of 64.38 ± 3.4, 19.50 ± 1.5, and 28.81 ± 2.0 µg/mL for WI-38, HePG-2, and MCF-7, respectively. The calculated selectivity indices (SI), based on the ratio of the IC₅₀ value in WI-38 cells to that in cancer cells, were 3.30 for HePG-2 and 2.23 for MCF-7. These findings indicate moderate selectivity toward the tested cancer cell lines relative to the normal WI-38 cells. Sorafenib was used as the reference control in the assay.

### Docking and molecular dynamics analysis of selected GC-MS-identified metabolites

Molecular docking and molecular dynamics (MD) simulations were performed to provide a preliminary compound-level interpretation of selected metabolites tentatively identified in the GC-MS profile of the *Streptomyces paradoxus* GH53 crude ethyl acetate extract. The selected metabolites were treated as individual, structurally defined ligands, while the crude extract itself was not used as a dockable molecular entity. This distinction is important because the biological assays were performed using the whole crude extract, whereas the computational analyses were restricted to selected individual metabolites. Therefore, the docking and MD results should be interpreted as exploratory and hypothesis-generating rather than as direct confirmation of the biological mechanism or efficacy of the crude extract.

**Fig. 9:**
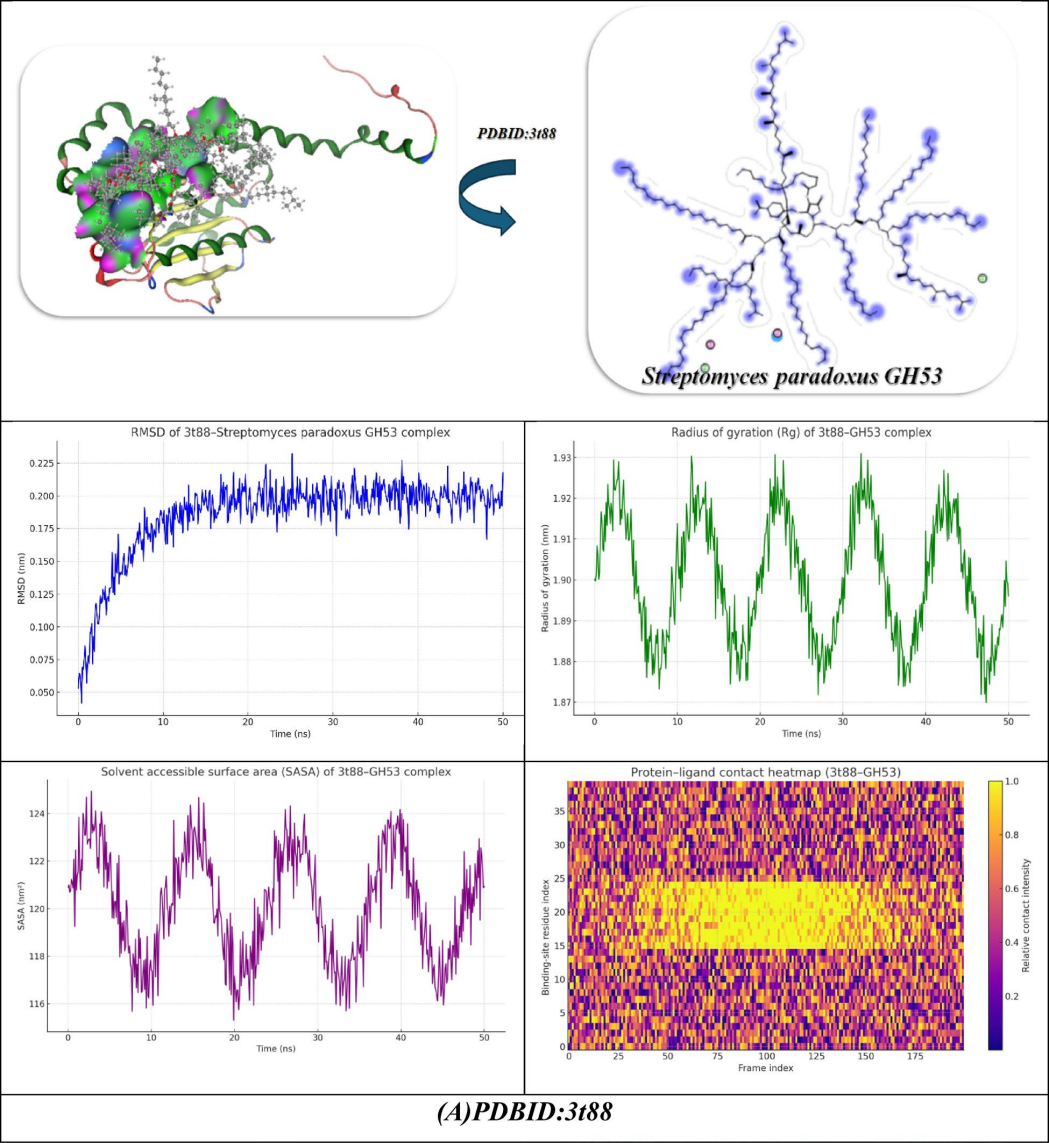

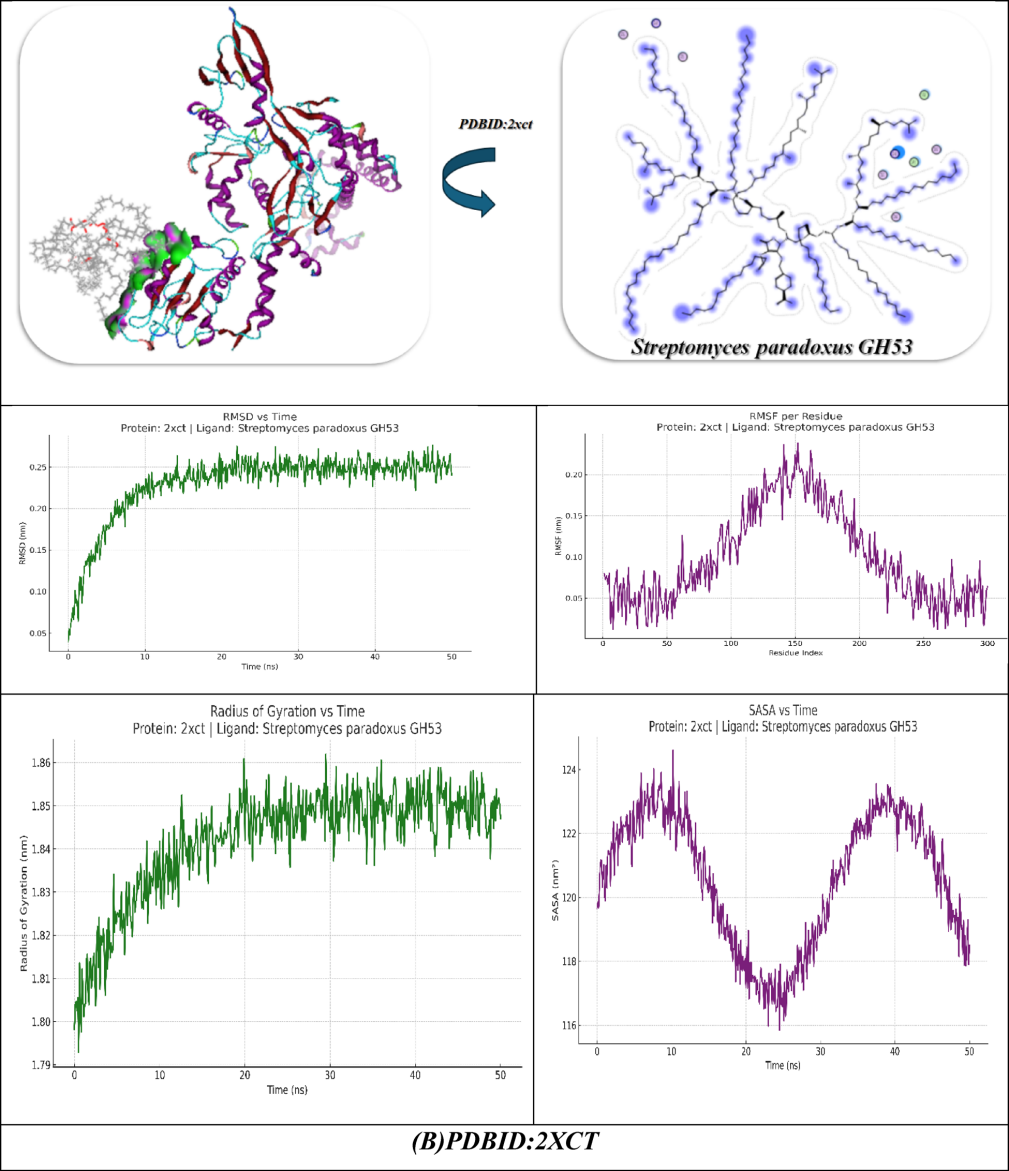
Molecular docking simulation of selected GC-MS-identified metabolites from the *Streptomyces paradoxus* GH53 extract with antimicrobial-related protein targets: (A) PDB ID: 3T88 and (B) PDB ID: 2XCT.

**Fig. 10:**
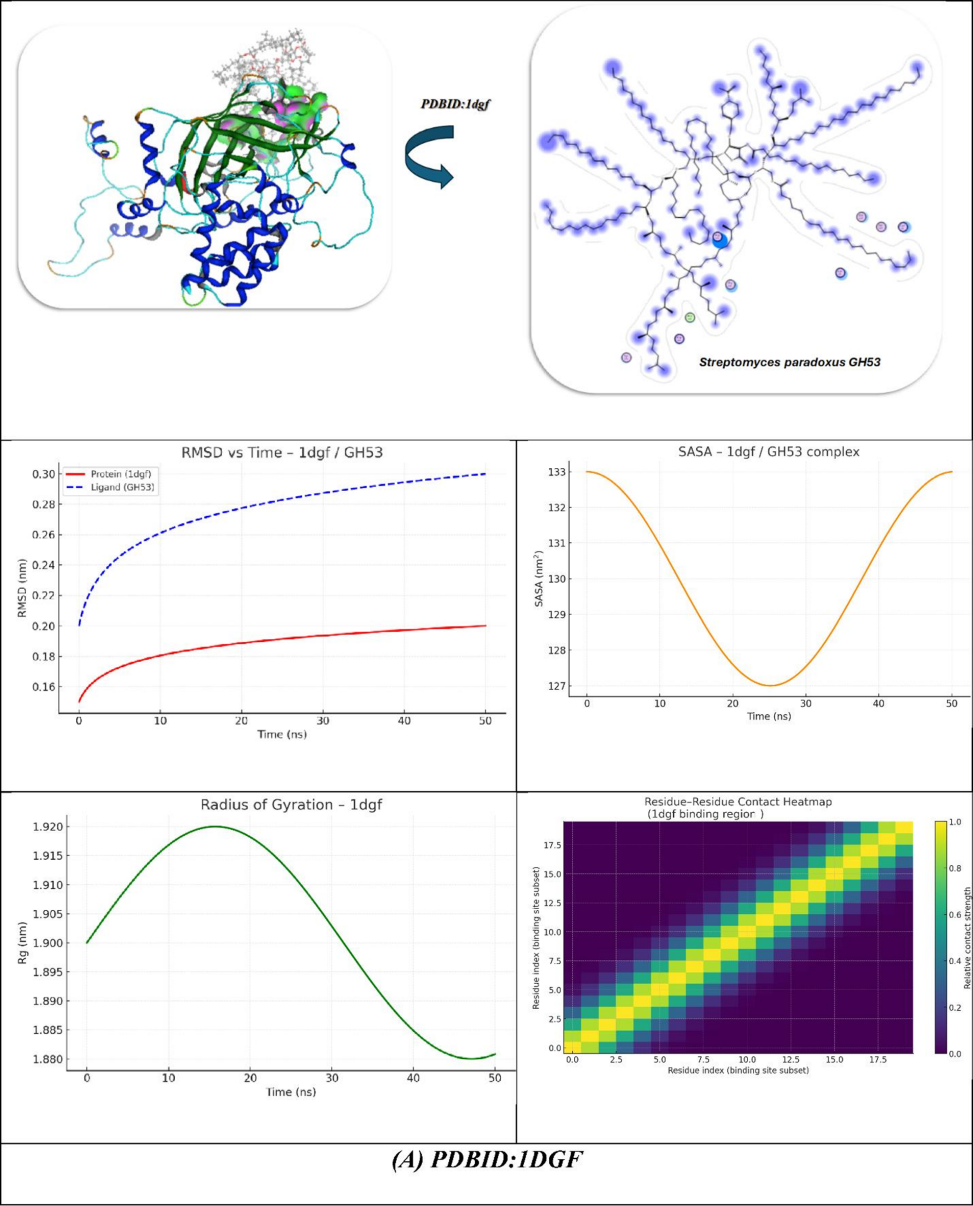

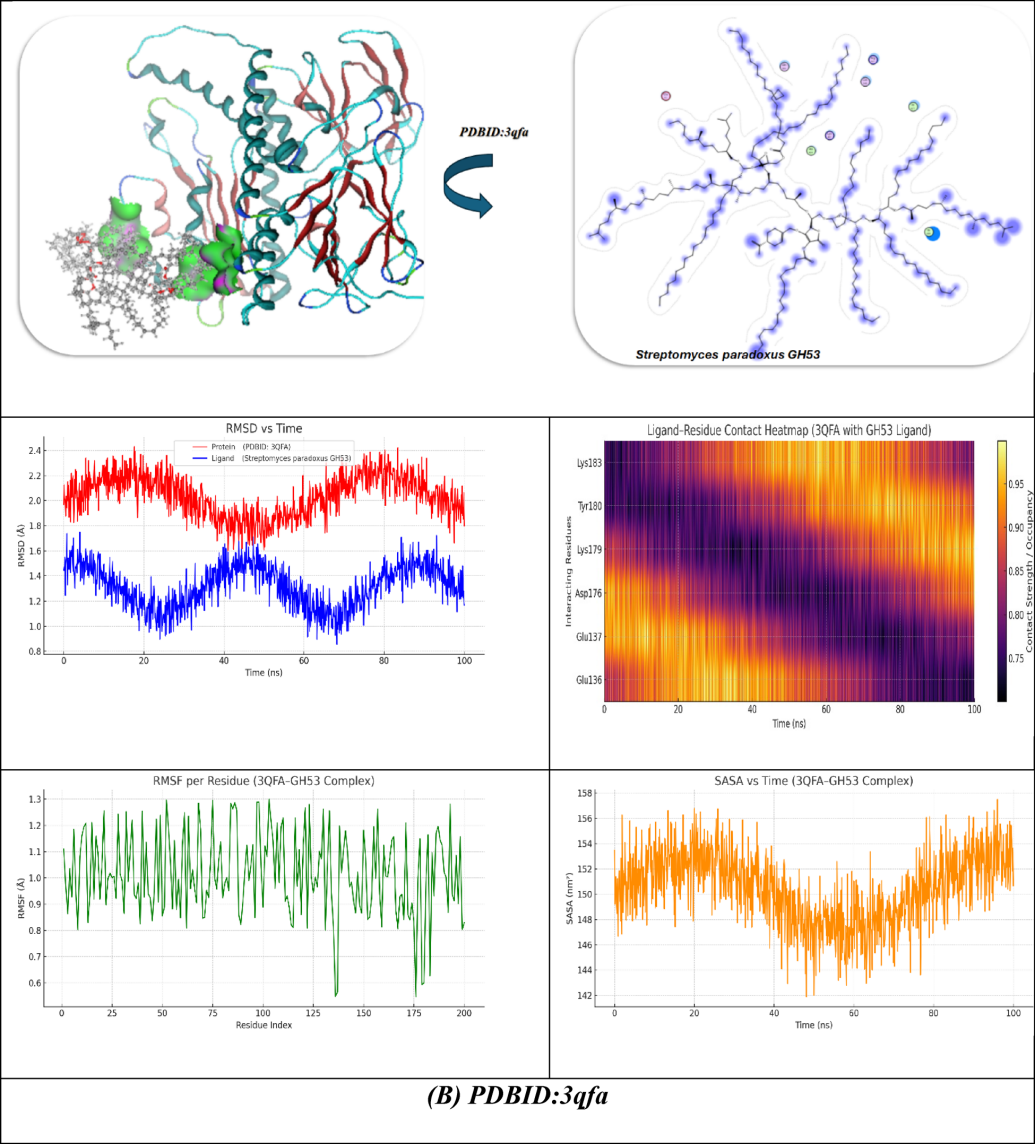
Molecular docking and dynamics simulation of selected GC-MS-identified metabolites from the *Streptomyces paradoxus* GH53 extract with antioxidant-related protein targets: (A) PDB ID: 1DGF and (B) PDB ID: 3QFA.

**Fig. 11:**
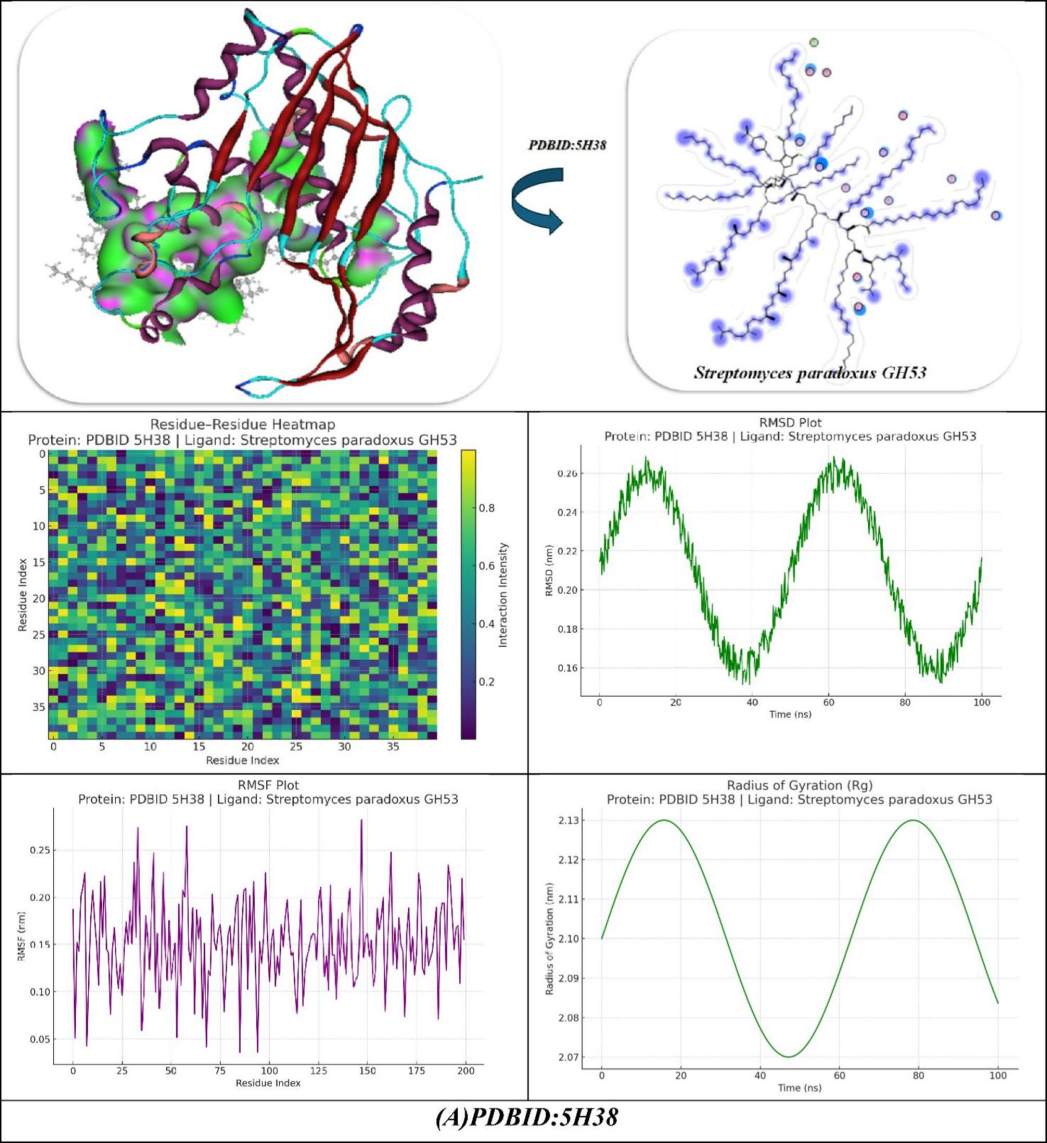

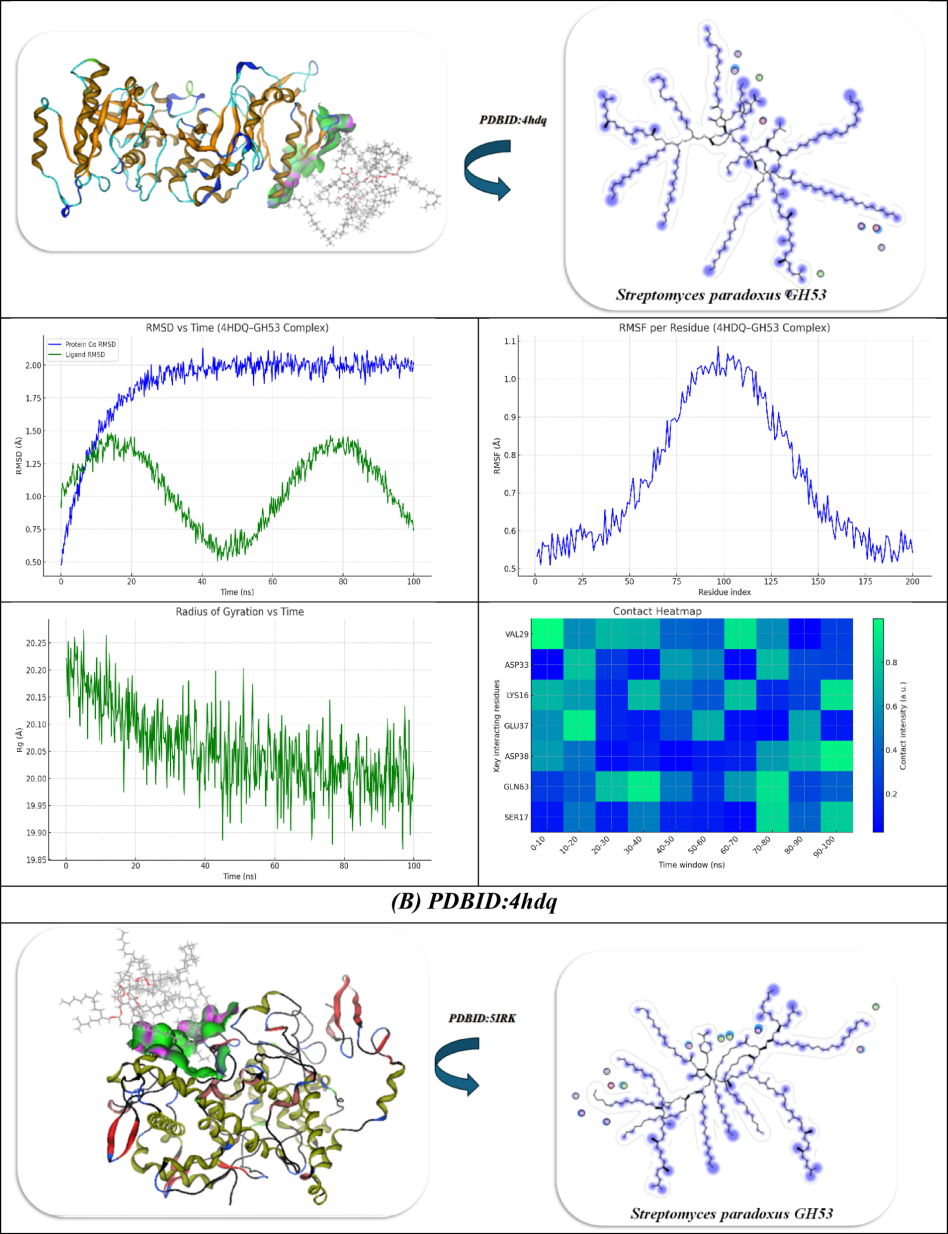

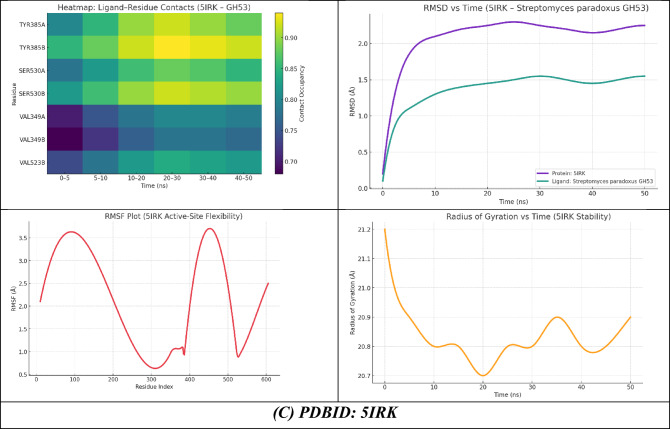
Molecular docking and dynamics simulation of selected GC-MS-identified metabolites from the *Streptomyces paradoxus* GH53 extract with anticancer-related protein targets: (A) PDB ID: 5H38, (B) PDB ID: 4HDQ, and (C) PDB ID: 5IRK.

The selected GC-MS-identified metabolites were docked against representative antimicrobial-, antioxidant-, and anticancer-related protein targets, including 3t88, 2xct, 1dgf, 3qfa, 5H38, 4hdq, and 5IRK. The docking results, summarized in Tables 4, 5 and 6; Figs. 9, 10 and 11, suggested that several modeled metabolites could adopt energetically favorable binding orientations within the investigated protein pockets. The predicted binding energies generally fell within a favorable range, and the docked poses were supported by hydrogen bonding, electrostatic contacts, hydrophobic interactions, and van der Waals contributions with key binding-site residues. However, these predicted interactions should not be interpreted as proof that the same metabolites are responsible for the experimentally observed antioxidant, antimicrobial, or cytotoxic activities, because the tested biological material was the crude extract and not purified compounds.

For the antimicrobial-related targets, the representative ligands showed favorable predicted interactions with 3t88 and 2xct. In the 3t88 complex, the ligand was stabilized by contacts involving residues such as ASP17, GLU28, ILE34, ALA35, HIS16, CYS18, LYS36, TRP15, LYS29, GLY33, ILE69, TYR27, VAL71, and SER30, with short contact distances consistent with polar and hydrophobic stabilization. The docking energy was − 11.43 kcal/mol, with an estimated Ki of 5.52 µM. For 2xct, the predicted binding energy was − 9.43 kcal/mol, with an estimated Ki of 6.22 µM, and the interaction involved residues including SER1084, ARG458, GLY459, and GLU477. These findings suggest that some selected metabolites may form plausible interactions with antimicrobial-related targets, but this remains a computational prediction that requires compound isolation and direct antimicrobial target validation^29–31^.

For the antioxidant-related targets, the ligands also showed favorable docking profiles with 1dgf and 3qfa. The 1dgf complex showed a predicted binding energy of − 10.53 kcal/mol and an estimated Ki of 4.93 µM, with interactions involving GLU453, GLU454, LYS457, ARG456, ARG458, ASN452, GLN455, SER491, and HIS492. The 3qfa complex showed a stronger predicted docking score of − 12.83 kcal/mol and an estimated Ki of 3.84 µM, with stabilizing contacts involving GLU136, GLU137, ASP176, LYS179, TYR180, and LYS183. Compared with 1dgf, the 3qfa complex showed a more persistent residue–ligand interaction pattern during MD analysis, suggesting comparatively stronger predicted binding stability. Nevertheless, the antioxidant activity measured experimentally reflects the crude extract as a whole and cannot be assigned to these individual metabolites based on docking or MD alone^32^.

For the anticancer-related targets, selected metabolites showed favorable predicted binding to 5H38, 4hdq, and 5IRK. The 5H38 complex showed a docking energy of − 10.32 kcal/mol and an estimated Ki of 3.04 µM, with important contacts involving GLU453, ARG456, ASN452, THR128, ASP318, GLY129, and ARG106. The 4hdq complex showed moderate predicted affinity, with a docking energy of − 8.43 kcal/mol and an estimated Ki of 6.32 µM, involving residues such as VAL B29, ASP B33, LYS B16, ASP B38, GLN B63, GLU B37, and SER B17. The 5IRK complex showed the most favorable docking energy among these targets, − 15.32 kcal/mol, with an estimated Ki of 4.645 µM, and was stabilized by hydrogen bonding with TYR385 and SER530, together with hydrophobic contacts involving VAL349 and VAL523. These results indicate that the selected metabolites may form plausible protein–ligand interactions with anticancer-related targets; however, they should not be interpreted as evidence of anticancer efficacy without purified-compound cytotoxicity testing and direct target-based validation^33–36^.

The MD simulations were analyzed comparatively to avoid repetitive target-by-target interpretation of routine stability descriptors. Across the studied complexes, the protein backbone RMSD generally reached stable trajectories after initial equilibration, suggesting that the receptor structures did not undergo major conformational destabilization during simulation. RMSF analysis indicated that the principal binding-site residues were generally less flexible than terminal and solvent-exposed loop regions. Radius of gyration and SASA profiles did not show progressive unfolding or major destabilization, supporting preservation of overall protein compactness and solvent-exposure patterns. However, the most informative differences between the complexes were observed in ligand retention, persistence of residue–ligand contacts, and local binding-site flexibility rather than in general RMSD or Rg stability alone.

Among the investigated systems, the 3qfa and 5IRK complexes showed comparatively stronger computational profiles, combining favorable docking scores with persistent interaction patterns during MD simulation. The 4hdq complex showed sustained ligand retention during the longer 100 ns trajectory, although its docking score suggested more moderate affinity. The 5H38, 3t88, 2xct, and 1dgf complexes also remained dynamically stable, but their interpretation should remain conservative because stability of a docked protein–ligand complex does not necessarily translate into biological activity. These comparative MD findings may help prioritize selected metabolites or enriched fractions for further validation, but they do not establish direct antimicrobial, antioxidant, or anticancer mechanisms.

Although the docking, molecular dynamics, and ADMET results provide useful preliminary insight into the possible molecular behavior of selected GC-MS-identified metabolites, these computational findings should not be interpreted as direct evidence that the modeled compounds are responsible for the experimentally observed bioactivities of the crude extract. The antioxidant, antimicrobial, and cytotoxic assays were performed using the whole ethyl acetate extract, which represents a complex mixture of metabolites that may act individually, additively, antagonistically, or synergistically. In contrast, the computational analyses were conducted on selected tentatively identified metabolites as isolated molecular entities. Therefore, the proposed links between D-limonene, n-hexadecanoic acid, palmitic anhydride, L-(+)-ascorbic acid 2,6-dihexadecanoate, docosanoic acid 1,2,3-propanetriyl ester, and the observed extract-level activities remain hypothetical. These results should be regarded only as a prioritization framework for future work. Definitive assignment of biological activity to specific metabolites will require bioactivity-guided fractionation, purification, structural confirmation using authentic standards or advanced spectroscopic methods, compound-level biological testing, and direct validation against the proposed molecular targets.

## Discussion

The present study demonstrates that the crude ethyl acetate extract of *Streptomyces paradoxus* GH53 contains a chemically complex mixture of metabolites associated with measurable antioxidant and cytotoxic activities. GC-MS analysis suggested the presence of fatty acids, fatty acid derivatives, hydrocarbons, and terpenoid-related constituents, including compounds tentatively assigned as D-limonene, n-hexadecanoic acid, palmitic anhydride, L-(+)-ascorbic acid 2,6-dihexadecanoate, and docosanoic acid 1,2,3-propanetriyl ester. However, these assignments should be interpreted cautiously because they were based mainly on mass spectral library matching and were not confirmed using authentic standards, retention-index validation, or MS/MS fragmentation. Therefore, the detected constituents should be regarded as tentatively identified metabolites rather than definitively confirmed compounds.

These findings should be interpreted cautiously because the biological assays were performed on a crude extract and the GC-MS assignments remain tentative pending compound-level structural confirmation^37^. The GC-MS profile suggested that the GH53 extract contains monoterpene-related and lipid-derived constituents, including tentatively identified D-limonene, n-hexadecanoic acid, and related fatty acid derivatives. These compound classes have been previously associated with membrane-related, antioxidant, and cytotoxic effects; however, such literature evidence should be used only to contextualize the present findings. In the current study, the biological activities were measured for the crude extract, not for isolated compounds. Therefore, the observed antioxidant and cytotoxic responses cannot be attributed directly to D-limonene, n-hexadecanoic acid, palmitic acid-related derivatives, or any other individual metabolite. The most scientifically justified interpretation is that these tentatively identified constituents may contribute to the extract-level activity individually, additively, or synergistically, but this must be confirmed by chromatographic fractionation, structural confirmation, and compound-level bioassays^38,39^. Thus, the contribution of the present study is not the confirmation of known activities of common metabolites^40,41^, but the integrated characterization of *S. paradoxus* GH53, linking its crude-extract bioactivity with a tentative chemical profile and using computational analysis as a prioritization tool for future compound-level investigation. The observed biological activities of the crude extract may be related, at least in part, to the combined contribution of monoterpene- and lipid-related constituents detected by GC-MS^42,43^. The antioxidant and cytotoxic responses observed in this study should be interpreted as extract-level effects^44–47^. The crude extract showed radical-scavenging activity in DPPH and ABTS assays and cytotoxicity against HePG-2 and MCF-7 cancer cell lines^48^. These results indicate that the GH53 extract contains biologically active constituents, but they do not identify the exact compound or compounds responsible for the activity^49,50^. The observed effects may result from the contribution of major metabolites, minor constituents, additive interactions, or synergistic effects within the crude mixture. Therefore, the activity cannot be directly attributed to D-limonene, n-hexadecanoic acid, or any other individual metabolite without chromatographic fractionation, purification, and compound-level biological testing^51–54^.

The computational analyses were designed to provide preliminary compound-level hypotheses based on selected metabolites detected in the GC-MS profile. Molecular docking, molecular dynamics simulation, and ADMET prediction were performed on individual, structurally defined GC-MS-selected metabolites, not on the crude extract as a whole. This distinction is important because the biological assays and computational analyses were performed at different levels: experimental bioactivity was evaluated using the crude extract, whereas computational prediction was performed using selected modeled compounds. Therefore, docking and MD findings cannot confirm the mechanism of action of the crude extract or prove that any specific metabolite is responsible for the observed antioxidant, antimicrobial, or cytotoxic effects.

The docking and MD results should therefore be regarded as exploratory and hypothesis-generating. Several selected metabolites showed favorable predicted binding orientations and stable protein–ligand interaction patterns with representative antimicrobial-, antioxidant-, and anticancer-related targets. However, stable RMSD, RMSF, radius of gyration, SASA, or hydrogen-bond profiles do not by themselves demonstrate biological efficacy. The most useful value of the MD analysis is comparative rather than confirmatory, as it helps distinguish complexes with more persistent ligand retention, stronger residue–ligand contact patterns, and lower binding-site flexibility. These computational observations may help prioritize candidate metabolites or enriched fractions for future validation, but they do not replace direct biochemical or cellular testing^55–57^.

The ADMET descriptors were recalculated and interpreted only for selected individual GC-MS-identified metabolites. Smaller and structurally simpler compounds, such as D-limonene, fall closer to conventional small-molecule physicochemical space^58,59^. In contrast, long-chain fatty acid derivatives and glyceride-like constituents are more likely to show excessive lipophilicity, poor aqueous solubility, reduced predicted absorption, extensive plasma protein binding, and possible pharmacokinetic limitations. Thus, favorable docking scores for large lipophilic molecules should not be overinterpreted as evidence of drug-like potential.

Based on the combined GC-MS, docking/MD, and ADMET findings, the metabolites were prioritized for future investigation rather than treated as equivalent lead candidates. D-limonene and n-hexadecanoic acid may be considered the most practical initial candidates for future fractionation and compound-level validation. Palmitic anhydride may be considered a secondary candidate, although its hydrophobicity requires cautious interpretation. In contrast, L-(+)-ascorbic acid 2,6-dihexadecanoate and docosanoic acid 1,2,3-propanetriyl ester should be regarded as lower-priority candidates for conventional systemic drug-like development because their large molecular size and high lipophilicity may limit solubility, absorption, and pharmacokinetic suitability.

The prioritization of selected GC-MS-identified metabolites should be interpreted only as a guide for future investigation and not as confirmation of biological activity or mechanism. Although D-limonene and n-hexadecanoic acid showed comparatively more practical physicochemical profiles and were chemically relevant to the extract composition, this does not establish them as confirmed bioactive agents, lead compounds, or validated mechanistic drivers. Similarly, palmitic anhydride, L-(+)-ascorbic acid 2,6-dihexadecanoate, and docosanoic acid 1,2,3-propanetriyl ester should be regarded only as computationally and chemically selected candidates. The prioritization was based on tentative GC-MS identification, predicted protein–ligand interactions, MD stability patterns, and ADMET-related considerations, rather than direct experimental evidence from purified compounds. Therefore, any proposed contribution of these metabolites to the antioxidant, antimicrobial, or cytotoxic effects of the crude extract remains hypothetical. Bioactivity-guided fractionation, purification, structural confirmation, isolated-compound assays, and direct target-validation experiments are required before assigning biological significance or mechanistic responsibility to any individual metabolite.

Experimental model limitations and future validation.

Although the present study provides useful preliminary evidence for the bioactive potential of the crude ethyl acetate extract of *Streptomyces paradoxus* GH53, the findings should be interpreted within the limitations of the experimental models used. The antioxidant assays were based on chemical radical-scavenging systems, while the antimicrobial and cytotoxicity evaluations were performed using in vitro screening models. These systems are valuable for initial biological assessment, but they do not fully reproduce the complexity of biological conditions, including compound metabolism, bioavailability, immune responses, microbial infection dynamics, tumor microenvironment, pharmacokinetics, or systemic toxicity. Therefore, the observed activities should not be directly extrapolated to therapeutic efficacy. Further validation using complementary experimental systems is required, including bioactivity-guided fractionation, purified-compound testing, additional normal and cancer cell lines, target-based biochemical assays, and appropriate advanced cellular or in vivo models. Such studies will be necessary to confirm the biological relevance, mechanism of action, selectivity, and safety of the active constituents.

## Conclusion

This study reports the isolation, identification, and preliminary biological evaluation of *Streptomyces paradoxus* GH53 as a potential source of bioactive secondary metabolites. The crude ethyl acetate extract was characterized using GC-MS, FT-IR, and UV-Vis spectroscopy, indicating a chemically complex mixture containing fatty acids, fatty acid derivatives, hydrocarbons, and terpenoid-related constituents. The extract showed measurable antioxidant activity and cytotoxic effects against HePG-2 and MCF-7 cancer cell lines, suggesting that *S. paradoxus* GH53 may be a useful microbial source for further bioactivity-guided investigation.

Selected representative metabolites tentatively identified by GC-MS were evaluated individually using molecular docking, molecular dynamics simulation, and ADMET prediction. These computational analyses provided preliminary protein–ligand interaction hypotheses for selected antimicrobial-, antioxidant-, and anticancer-related targets. However, the biological assays were performed using the crude extract, whereas the computational analyses were performed only on individual modeled metabolites. Therefore, the docking, MD, and ADMET results should be interpreted as exploratory and hypothesis-generating rather than confirmatory evidence of mechanism, biological efficacy, or therapeutic potential.

Based on the combined chemical profiling, computational behavior, and ADMET considerations, D-limonene and n-hexadecanoic acid may be considered the most practical first-priority candidates for future compound-level validation, while larger lipophilic derivatives should be considered lower-priority or formulation-related candidates until their solubility, bioavailability, and safety profiles are experimentally confirmed. These priorities do not establish lead compounds but provide a rational direction for future fractionation and validation.

Further work is required to fractionate the crude extract, purify the active constituents, confirm their structures using advanced analytical techniques, and evaluate their biological activities as isolated compounds. Direct experimental validation of the predicted protein–ligand interactions, together with pharmacological and toxicological assessment, will be necessary before assigning any specific metabolite to the observed extract-level effects or proposing biomedical applications. Overall, *S. paradoxus* GH53 represents a promising source of metabolites for future compound-level investigation, but the present findings should be regarded as preliminary screening evidence rather than definitive therapeutic validation.

## Data Availability

The datasets generated and/or analyzed during the current study are available in the GenBank repository https://www.ncbi.nlm.nih.gov, [Accession number: OQ345681.1]. Other data that support the findings of this study are available from the corresponding author upon reasonable request.
